# Deferoxamine-Modified Hybrid Materials for Direct Chelation of Fe(III) Ions from Aqueous Solutions and Indication of the Competitiveness of *In Vitro* Complexing toward a Biological System

**DOI:** 10.1021/acsomega.1c01411

**Published:** 2021-06-03

**Authors:** Mateusz Pawlaczyk, Grzegorz Schroeder

**Affiliations:** Faculty of Chemistry, Adam Mickiewicz University, Uniwersytetu Poznańskiego 8, Poznań 61-614, Poland

## Abstract

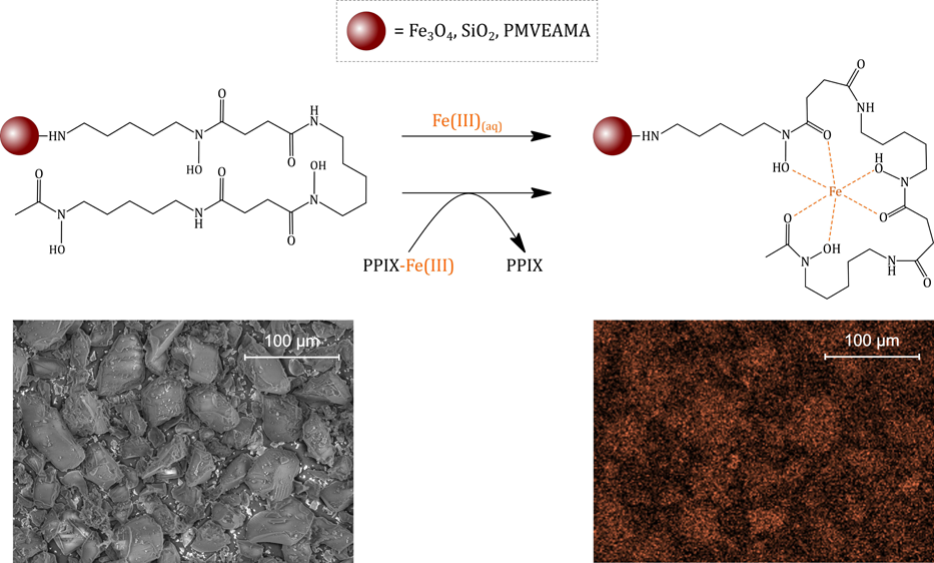

Deferoxamine (DFO) is one of the most potent iron ion complexing agent belonging to a class of trihydroxamic acids. The extremely high stability constant of the DFO–Fe complex (log β = 30.6) prompts the use of deferoxamine as a targeted receptor for scavenging Fe(III) ions. The following study aimed at deferoxamine immobilization on three different supports: poly(methyl vinyl ether-*alt*-maleic anhydride), silica particles, and magnetite nanoparticles, leading to a class of hybrid materials exhibiting effectiveness in ferric ion adsorption. The formed deferoxamine-loaded hybrid materials were characterized with several analytical techniques. Their adsorptive properties toward Fe(III) ions in aqueous samples, including pH-dependence, isothermal, kinetic, and thermodynamic experiments, were investigated. The materials were described with high values of maximal adsorption capacity *q*_m_, which varied between 87.41 and 140.65 mg g^–1^, indicating the high adsorptive potential of the DFO-functionalized materials. The adsorption processes were also described as intense, endothermic, and spontaneous. Moreover, an exemplary magnetically active deferoxamine-modified material has been proven for competitive *in vitro* binding of ferric ions from the biological complex protoporphyrin IX–Fe(III), which may lead to a further examination of the materials’ biological or medical applicability.

## 1 Introduction

Iron ions as microelements play a significant role in the stimulations of various functions in human organisms. Several health disorders may lead to an increased level of nontransferrin bound iron (NTBI), which might accumulate in healthy tissues, causing several dysfunctions, such as cardiac, hepatic, or pancreatic diseases. Burst release of iron also occurs during subarachnoid hemorrhage (a devastating subtype of stroke), which leads to hemoglobin breakdown, causing serious oxidative injuries and neuronal death. Moreover, iron excess promotes the formation of reactive oxygen species (ROS), which may oxidize various cell components such as lipid membranes, nucleic acids, or protein. Iron overload may also trigger a more rapid proliferation of iron-demanding cancer cells.^1−3^

Among many classes of domains responsible for effective chelation of metal ions, siderophores are the ones that bind iron selectively or exhibit extremely high binding constants. Deferoxamine (DFO) is a siderophore belonging to a class of trihydroxamic acids and is naturally secreted by bacterium species *Streptomyces pilosus*. Deferoxamine as a hexadentate molecule coordinates iron ions in a ratio of 1:1 with an extremely high stability constant β at a level of 4.0 × 10^30^. Its complexes with other metal ions are formed with much lower stability constants. This property has prompted deferoxamine-mediated ion overload treatment for many years of clinical therapy.^4−7^ Moreover, free deferoxamine exhibits beneficial therapeutic effects, such as antifibrotic effects, protection against acetaminophen-induced liver injuries, or inhibition of neurodegenerative Alzheimer’s and Huntington’s diseases.^8−11^

Deferoxamine is being used in clinical treatment; however, its application is limited due to its poor *in vivo* absorption to the gut, rapid renal excretion causing short plasma half-time, and sunlight hypersensitivity, which leads to enhanced production of ROS.^1,12^ Thus, several immobilization and functionalization approaches to incorporate different DFO formulations for analytical and biochemical applications have been investigated. Good pharmacokinetic parameters and improved bioapplicability were proven for deferoxamine conjugates with various adamantane derivatives,^13^ reverse emulsion nanogels containing DFO and glycine,^14^ a poly(d,l-lactide) membrane modified with DFO,^15,16^ or a synthesized c(RGDfK)–DFO–^89^Zr system.^17,18^

DFO properties has also prompted a design of functional materials dedicated to adsorption or sensing of Fe(III) ions. The implemented DFO-functionalized materials were based on, e.g., mesoporous silica MCM-41,^19,20^ Sepharose gel,^21^ or filtration paper Whatman,^22^ leading to biocompatible materials for direct Fe(III) sensing in aqueous or biological samples, using classic analytical techniques. Interestingly, a few reports aimed at application of a new approach for quantification of the amount of ferric ions chelated by DFO-functionalized materials, which involved a detection of Fe–O band signal intensities in FT-IR or surface-enhanced Raman scattering (SERS) spectra.^23−25^

The following research aimed to synthesize a series of deferoxamine-functionalized hybrid materials based on three different supports: poly(methyl vinyl ether-*alt*-maleic anhydride) (PMVEAMA), silica microparticles, and magnetite nanoparticles, which were implemented as ferric ion scavengers. The characterized materials were subjected to studies of their adsorptive properties toward Fe(III) ions, including a sequence of pH-dependence, isothermal, kinetic, and thermodynamic studies. The comprehensive studies led to several parameters describing the materials’ applicability for the metal binding, such as the most effective adsorption environment, materials’ adsorption capacities, rates of the adsorbate binding, or thermal coefficients. Moreover, the exemplary magnetite-based material was considered for competitive chelation of ferric ions from a biological complex of protoporphyrin IX (PPIX) and Fe(III) ions, which corresponds to a naturally occurring complex – hemin. The description of an adsorptive potential of the materials and characterization of their *in vitro* application toward competitive chelation of ferric ions may lead to a new class of eco-friendly and biocompatible adsorbents finding application in biomedical science.

## 2 Results and Discussion

The designed deferoxamine-functionalized hybrid materials were synthesized, characterized with several analytical techniques, and subsequently subjected to adsorption of Fe(III) ions from aqueous solutions to establish an influence of the support used on the adsorptive properties of the materials. Predominantly, the matrices’ size and functionalization potential would have had the most impact on the materials’ adsorption efficiency, which has been investigated in the following article.

### 2.1 Synthesis of Deferoxamine-Functionalized Hybrid Materials

**Figure 1:**
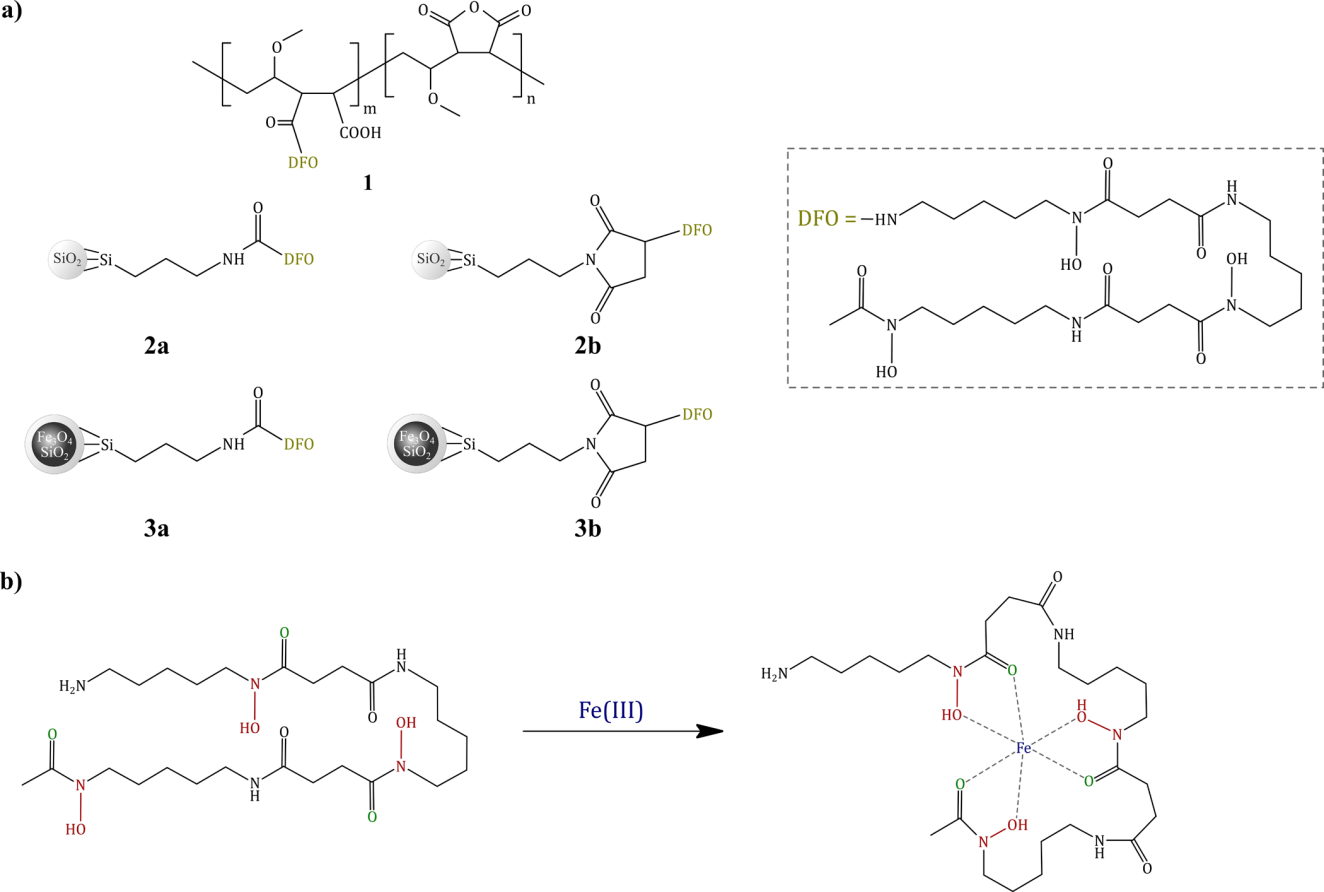
(a) Structures of the synthesized deferoxamine-functionalized hybrid materials; (b) formation of the Fe(III)–deferoxamine complex.

The designed adsorbents consisted of three different supports, which were biocompatible polymeric chains of poly(methyl vinyl ether-*alt*-maleic anhydride) (PMVEAMA), commercially available amorphous silica microparticles functionalized with surface isocyanate and maleimide groups, and synthesized Fe_3_O_4_ nanoparticles encapsulated within the silica matrix, which underwent functionalization with deferoxamine via isocyanate– and maleimide–silyl linkers. The functionalization strategy was based on a reaction between a terminal free amine group of deferoxamine with reactive pendant groups on the supports’ surface. Functionalization of PMVEAMA was afforded by maleic anhydride ring opening at elevated temperature under nucleophilic attack of the deferoxamine amine group. For both silica and Fe_3_O_4_, the attachment of deferoxamine was performed either by amine group addition to a highly electrophilic carbon atom of pendant isocyanate or by Michael addition of the amine group to carbon–carbon double bond of the maleimide ring. Accordingly, five hybrid materials were obtained, which structures are collected in Figure 1, and the synthetic routes are presented in Figure S1.

The Fe_3_O_4_ nanoparticles were obtained by coprecipitation from an aqueous solution containing Fe(III):Fe(II) salts in a ratio of 2:1 under alkaline conditions (pH ∼10).^26^ The synthesized magnetite nanoparticles were subsequently covered with a SiO_2_ layer, which was achieved by condensation of tetraethyl orthosilicate (TEOS) under alkaline conditions in a water/ethanol mixture. The obtained Fe_3_O_4_/SiO_2_ particles were then treated as a starting material for obtaining the Fe_3_O_4_-based hybrid materials. An introduction of the deferoxamine domain onto Fe_3_O_4_/SiO_2_ platform was achieved through two different linkers: isocyanate– and maleimide–silyl linkers. The isocyanate linker was reacted with a solution of deferoxamine in DMF under a N_2_ atmosphere, and the resulting silyl derivative of deferoxamine was incorporated into the silica matrix of Fe_3_O_4_/SiO_2_ material (material **3a**). The pre-synthesized maleimide linker (3-maleimide-propyltriethoxysilane) was anchored to a magnetite-based support, prior to reaction with deferoxamine, obtaining material **3b**. In the case of PMVEAMA–deferoxamine (material **1**) preparation, a suspension of PMVEAMA in toluene was added to a solution of deferoxamine in DMF at temperature of ∼110 °C, which led to a full opening of maleic rings in the polymer chain. Moreover, silica-based materials were synthesized by adding isocyanate- or maleimide-functionalized silica particles to deferoxamine solution in DMF at room temperature, yielding materials **2a** and **2b**, respectively.

### 2.2 Characterization of the Deferoxamine-Modified Adsorbents

**Figure 2:**
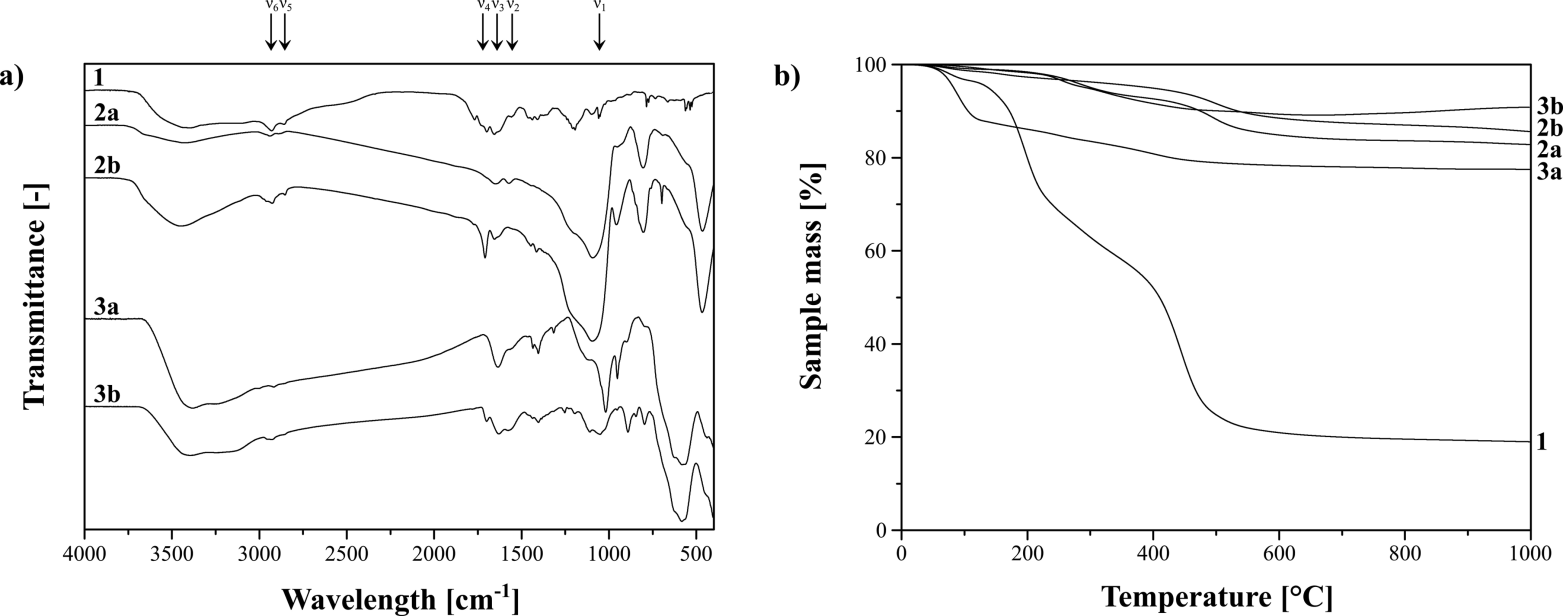
(a) FT-IR spectra of the adsorptive materials with indicated specific bands: ν_1_ = 1051 cm^–1^; ν_2_ = 1570 cm^–1^; ν_3_ = 1640 cm^–1^; ν_4_ = 1705 cm^–1^; ν_5_ = 2855 cm^–1^; ν_6_ = 2930 cm^–1^; (b) the thermogravimetric curves obtained during thermal analysis of the materials.

Each synthesized adsorbent was characterized with FT-IR spectroscopy, which spectra are collected in Figure 2a. The successful incorporation of deferoxamine into the supports’ surface is unambiguously proven by a band at 1051 cm^–1^ (ν_1_), which is related to N–OH stretching, specific for DFO structure.^27^ Such a signal is visible only in the spectrum of **1**, which is due to its overlapping by a broad band originating from Si–O–Si stretching of the silica matrix in each of the other materials. Nevertheless, signals at approximately 1570 and 1640 cm^–1^ (ν_2_ and ν_3_, N–H_(amide)_ and C=O_(amide)_, respectively) undoubtedly prove the formation and incorporation of amide bonds, and thus the presence of deferoxamine domains. Moreover, two bands at around 2855 and 2930 cm^–1^ (ν_5_ and ν_6_, respectively) are related to C–H stretching of methylene groups present in the deferoxamine structure. A signal at approximately 1705 cm^–1^ (ν_4_) on the spectra of **1**, **2b**, and **3b** may be attributed to the remaining unmodified domains, such as C=O stretching of maleic anhydride of PMVEAMA or C=C stretching of the maleimide ring in materials **2b** and **3b**. Each of the materials was also characterized using thermogravimetric measurements (Figure 2b). The very first step at a temperature range between 70 and 130 °C is strictly connected with the evaporation of solvent residues. For all the curves, the main decomposition step starts at approximately 150 °C, which corresponds to the melting point of deferoxamine. The TG curves of the materials based on either Fe_3_O_4_ or Fe_3_O_4_/SiO_2_ platforms exhibit this oxidation step with ∼7.5% loss of mass, corresponding to ∼0.135 mmol g^–1^ loading of deferoxamine. However, the spectrum of material **1** presents much more intensified sample decomposition by ∼25%, indicating the higher deferoxamine loading to PMVEAMA chains. Further decomposition steps present in the spectrum are connected with the oxidation of organic residues remaining unmodified by deferoxamine. Also, elemental analysis in CHN mode was performed for the synthesized hybrid materials, which results are collected in Table 1. The most informative values are nitrogen percentages in the samples, since nitrogen atoms appear only in deferoxamine domains and the maleimide linker, in which grafting is known, and therefore eliminates any calculation disturbances. Using the obtained %N values, the loading of the Fe-chelator on the supports was determined, which is in good agreement with the results obtained during thermal analysis.

**Table 1:** Values of Nitrogen, Carbon, and Hydrogen Percentages in the Adsorbents Obtained in Elemental Analysis with an Indication of the Calculated Deferoxamine-Loading Values

		elemental analysis			
adsorbent		% N	% C	% H	loading [mmol g^–1^]
**1**	PMVEAMA–DFO	7.04	51.96	8.51	1:3^a^
**2a**	SiO_2_–NCO–DFO	2.01	7.84	1.97	0.226
**2b**	SiO_2_–maleimide–DFO	1.31	8.29	1.72	0.134
**3a**	Fe_3_O_4_/SiO_2_–NCO–DFO	1.34	4.27	1.38	0.137
**3b**	Fe_3_O_4_/SiO_2_–maleimide–DFO	1.31	3.58	0.97	0.134

a For the polymer (PMVEAMA) functionalization, a ratio of modified to unmodified maleic anhydride domains was only calculated.

The results of XRD analysis of magnetite nanoparticle-based materials **3a** and **3b** are shown in Figure S2. The deferoxamine-modified materials’ spectra show no significant changes with respect to the spectrum of pristine Fe_3_O_4_ nanoparticles. This is related to incorporating a thin layer of silica–deferoxamine on their surface, which does not influence the spectrum shape. However, the hybrid materials’ spectra exhibit a broad peak of low intensity at around 21.1° related to the silica shell (even if shifted in relation to the theoretical pattern). Nevertheless, a signal at around 35.5° appears to be narrower in the **3a** and **3b** spectra than for bare Fe_3_O_4_, implying the material size increase. Signals at approximately 30.2° are slightly wider for the spectra of the functionalized hybrid materials, which is caused by the overlapping of Fe_3_O_4_ and SiO_2_ reflexes, both appearing at around 30.2°. Moreover, the hybrid materials’ spectra show a tiny reflex at around 36.4°, indicating the new organo-derivative XRD signal. On the basis of the positions of signals and their full width at half-maximum (FWHM) values, the mean size of the characterized materials *D_hkl_* was calculated using the Scherrer equation, which mathematical expression is given below, where *k* is the Scherrer constant [−], λ is the wavelength of X-ray irradiation [nm], *B* is the FWHM value [rad], and 2θ is the signal position [°]:
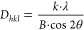
Accordingly, the calculated mean size of pristine Fe_3_O_4_ nanoparticles was 15.39 nm, while the mean sizes of materials **3a** and **3b** were 18.31 and 18.92 nm, respectively, which indicates a proper silyl–deferoxamine grafting, leading to the particles’ size increase.

**Figure 3:**
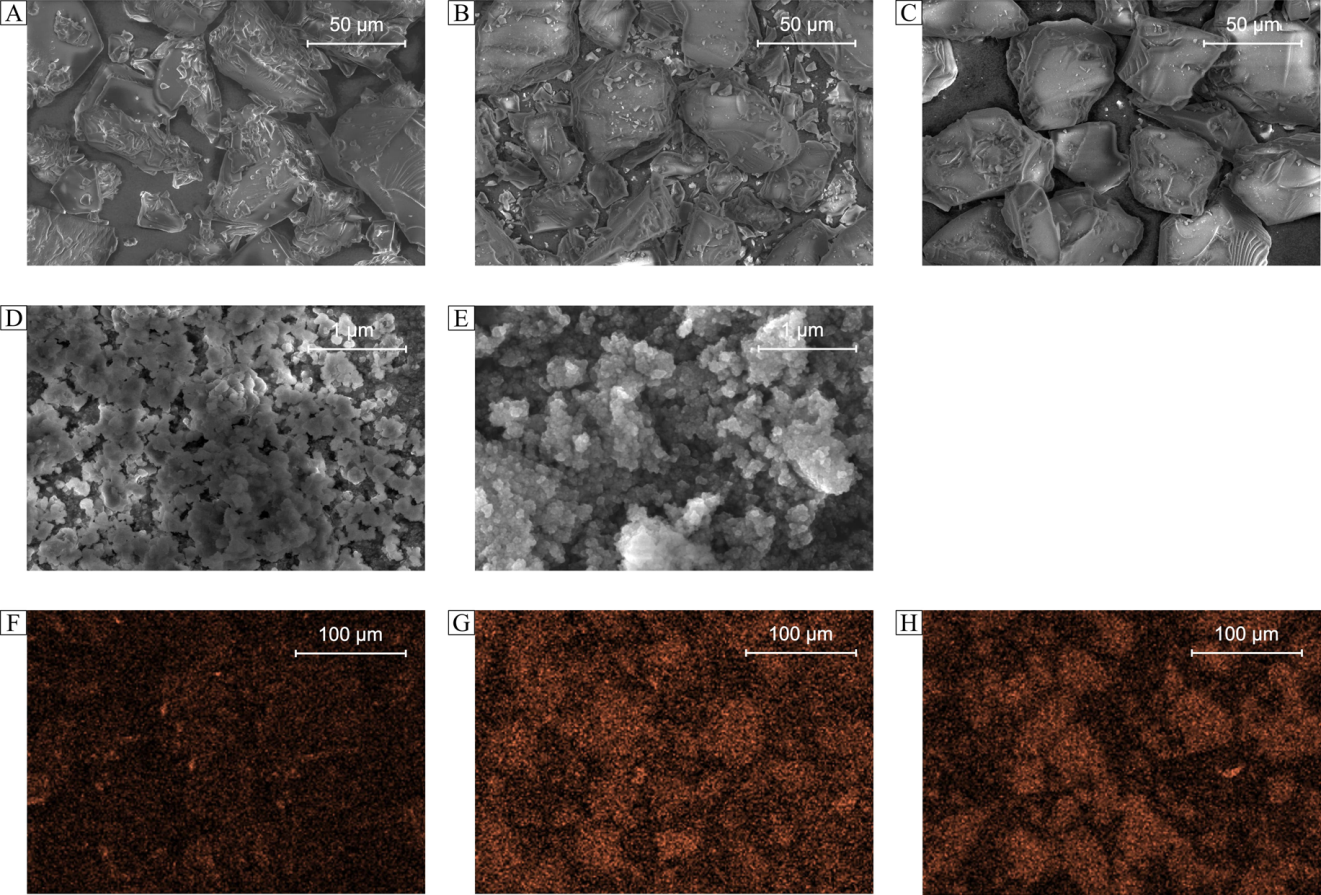
SEM images (A–C) and EDX–SEM mapping of Fe(III) ions (F–H) adsorbed to the hybrid materials: (A, F) PMVEAMA–DFO; (B, G) SiO_2_–NCO–DFO; (C, H) SiO_2_–maleimide–DFO). SEM images of magnetite-based hybrid materials: (D) Fe_3_O_4_/SiO_2_–NCO–DFO; (E) Fe_3_O_4_/SiO_2_–maleimide–DFO). Fe is visualized in orange.

All the obtained deferoxamine-loaded hybrid materials were also subjected to visualization using the SEM technique (Figure 3A–E). For the polymer-based and SiO_2_-based materials, the size of the particles is approximately 50 μm, which is connected with the polymeric character of the material’s **1** support, as well as with the size of bare silica particles used for the preparation of materials **2a** and **2b**, which was between 40 and 63 μm. Therefore, the size of the silica particles after functionalization with DFO residues might have insignificantly increased. The obtained materials, which are not based on the magnetite core, were also characterized with EDX–SEM after their treatment with Fe(III) ions. The Fe-mapping is presented in Figure 3F–H, which undoubtedly indicates the complexation of ferric ions in a higher extent by silica-based materials than the polymeric material. Such a phenomenon may be connected with a fixed porosity of silica particles, enhancing the ions’ adsorption efficiency. Moreover, Figure 3D,E presents the images of Fe_3_O_4_-based hybrid materials, which show the nanometric size of round-shaped particles of the deferoxamine-modified materials. The synthesized Fe_3_O_4_ nanoparticles of 15.39 nm as a magnetically active support did not significantly increase after encapsulation within the silica matrix and DFO conjugation, which is an important issue for nanomaterials applied as adsorbents.

The chelation of Fe(III) ions by deferoxamine domains may also influence the surface and the size of the pores of the materials. Thus, the most widely used techniques, which allow for assessment of structural features of solids – the BET (Brunauer, Emmett, and Teller) isotherm and the BJH (Barrett, Joyner, and Halenda) method – were implemented for material **3a**, as an example of the synthesized hybrid material. The used methods may highlight the differences between the porous features of the deferoxamine-loaded material before and after Fe adsorption. The molecular receptor chelates the cations by wrapping around them; therefore, it may influence the materials’ pore sizes and surface area. The porous properties of both material **3a** and Fe-loaded material **3a** were determined using N_2_ adsorption–desorption analysis, which is presented in Figure S3. The shapes of the isotherms for both bare and iron-loaded material **3a** can be classified as type IV, which postulates capillary condensation of the adsorbed gas in small pores at pressures below the saturation pressure of the gas. Therefore, based on the isotherms’ shapes, the material meets the criteria of mesoporosity.^28^ The material’s mesoporosity was also proven by BJH calculation during adsorption and desorption of nitrogen, which responded in the pore sizes of **3a** and **3a–Fe(III)** of 11.370 and 11.407 nm, respectively, calculated based on the adsorption curves, and 13.186 and 13.174 nm, respectively, for the desorption curves. Moreover, the volume of pores was established with mean values of 0.234 cm^3^ g^–1^ for material **3a** and 0.239 cm^3^ g^–1^ for Fe-loaded material **3a**. The surface areas of both materials were established using the BJH method, which gave 68.3 m^2^ g^–1^ for material **3a** and 66.7 m^2^ g^–1^ for material **3a** with chelated Fe(III) ions. All the parameters calculated for the two types of materials are very similar, with no drastic differences, which indicates that the formed deferoxamine–Fe(III) complex on the material’s surface has no significant impact on the porosity. Moreover, the second synthesized magnetite-based material (material **3b**) was characterized for its porous features using the same analytical methods. The material was described with its pore sizes of 11.402 and 13.079 nm calculated from the adsorption and desorption curves, respectively. Also, the surface area was calculated to be 68.3 m^2^ g^–1^ and the mean pore volume was calculated to be 0.279 cm^3^ g^–1^, which jointly indicate the similarity of both obtained materials containing the Fe_3_O_4_ core. Additionally, the pore size distributions established for the pristine magnetic materials and the one complexed with Fe(III) ions are presented in Figure S4. The distribution profiles obtained for materials **3a** and **3b** are almost overlapped, while the pore size distribution of the material **3a–Fe(III)** complex exhibits only insignificant change, which is consistent with the presented BET analysis.

All the materials were tested for their stability in paraphysiological conditions of phosphate-buffered saline (PBS). After the incubation, the solutes were analyzed using ESI–MS in order to investigate whether deferoxamine dissociates from the materials under the conditions mimicking the biological environment. The spectra of the solutes showed no signals referring to the dissociated deferoxamine residue (*m*/*z* 561.5), but only the signals corresponding to the components of the buffer used; therefore, the materials’ stability can be concluded.

### 2.3 Investigation of the Adsorptive Properties of the Fe-Chelating Materials

The synthesized materials were designed as chelating systems dedicated to Fe(III) ions since surface-introduced deferoxamine exhibits high binding efficiency. The formation of the deferoxamine–Fe complex was proven by ESI–MS measurements, which spectra are presented in Figure S5. An aqueous solution of free deferoxamine mesylate gives a single monoprotonated signal at 561.5 *m*/*z*, which is a molecular peak of deferoxamine. The spectra of its complexes with either Fe(III) or Fe(II) ions are presented in Figure S5b,c, respectively. Two signals corresponding to mono- and diprotonated complexes are visible at 614.4 and 307.7 *m*/*z*, respectively. Moreover, the signal present at 561.5 *m*/*z* related to free deferoxamine can be a result of electrospray ionization mode, which leads to easier fragmentation. Interestingly, the signal at 561.5 *m*/*z* is significantly lower for Fe(III)-complex than for Fe(II)-complex, which highlights the higher affinity of ferric ions toward the formation of DFO–iron complexes. The choice of different supports for anchoring deferoxamine (polymeric chain, amorphous silica, and SPIONs) can also lead to conclusions on their influence on the final adsorptive properties. To fully characterize the materials’ sorptive nature, several experiments were carried out, including isothermal, kinetic, and thermodynamic studies.

#### 2.3.1 Influence of pH on Fe(III) Adsorption

**Figure 4:**
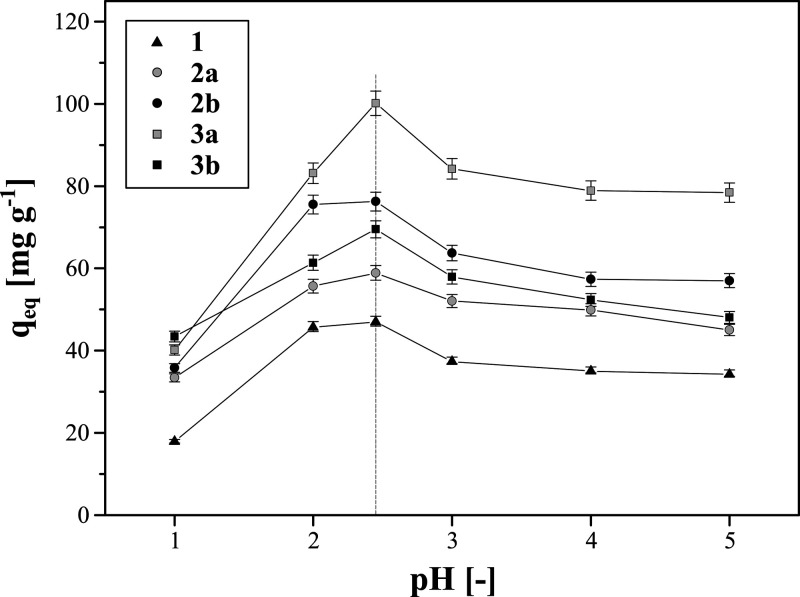
Influence of the solution pH on the amount of Fe(III) ions adsorbed on the deferoxamine-functionalized hybrid materials (gray dotted line corresponds to the pH of 5 mM aqueous solution of Fe(ClO_4_)_3_ – 2.45).

Figure 4 shows the dependence between Fe(III) ions adsorption efficiency on the hybrid materials and the solution pH. The materials were subjected to adsorption of ferric ions in the pH ranging between 1 and 5, according to precipitation of Fe(OH)_3_ in more basic conditions for 5 mM solution, as well as in 5 mM solution in distilled water, which was characterized to be of pH 2.45 (gray line in Figure 4). Below pH 6, the iron–deferoxamine complexes can be classified as [FeLH]^+^, where L is the ligand (deferoxamine), Fe is the ferric ion, and H is the proton.^29^ Therefore, the adsorption may be limited only due to repulsive interactions between excessive H^+^ and Fe^3+^ ions visible at pH 1. At the most acidic environment studied, the adsorption rates reached 38–62% of the maximal adsorption capacity under the given conditions. The adsorption rates increased with increasing pH, reaching maxima at pH 2.45, which corresponds to Fe(III) solution in pure distilled water. Then, *q*_eq_ values slightly decreased, primarily due to the use of sodium salts as buffers’ ingredients, leading to the competitive binding of Na^+^ ions. Nevertheless, the decrease is not drastic, ranging between 21.7 and 30.8%.

#### 2.3.2 Preparation of Adsorption Isotherms

**Figure 5:**
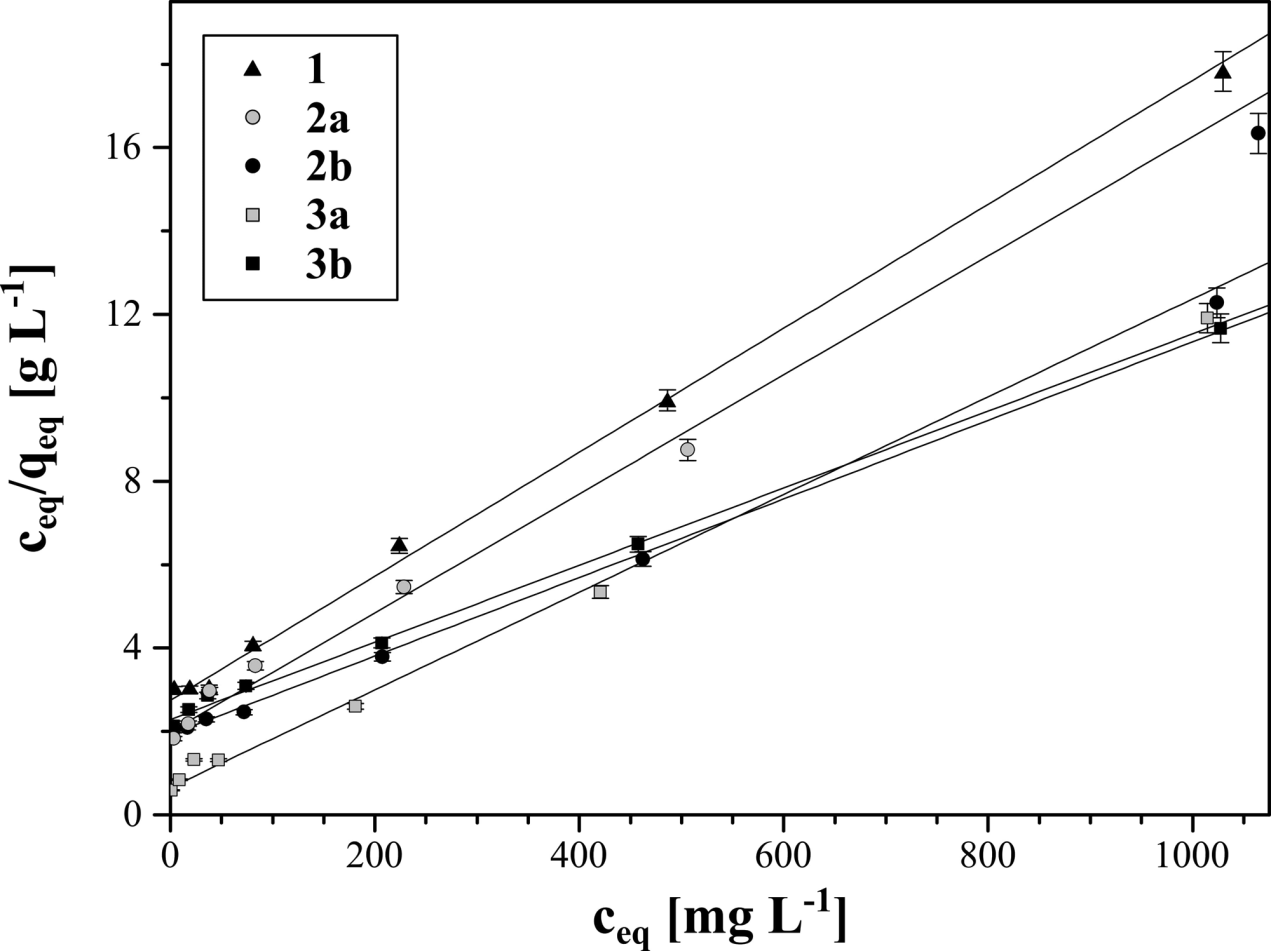
Fitting of the experimental data to the Langmuir isotherm model.

The experimental data obtained for adsorption isotherms were fitted to the Langmuir and the Freundlich models. The first model assumes a formation of the adsorbate monolayer on the adsorbent surface, which is due to the equal binding efficiency of all the binding sites and neglecting the interactions between adsorbed molecules, while the latter is mostly based on the assumption that adsorbate molecules may interact with each other via electrostatic, hydrogen, or π–π interactions, forming the adsorbate multilayer.^30^ Graphical representations of the Langmuir and the Freundlich isotherms are presented in Figure 5 and Figure S6, respectively, while the calculated parameters for both isothermal models are collected in Table 2.

**Table 2:** Isothermal Parameters Calculated for Fe(III) Adsorption on the Hybrid Materials

	Langmuir isotherm				Freundlich isotherm			
adsorbent	*q*_max_ [mg g^–1^]	*K*_L_ [×10^2^] [L mg^–1^]	*R*^2^	χ^2^	1/*n* [−]	*K*_F_ [mg g^–1^ (L mg^–1^)^1/*n*^]	*R*^2^	χ^2^
**1**	87.41 ± 2.20	0.60 ± 0.03	0.9984	0.120	0.79 ± 0.01	0.64 ± 0.02	0.9709	0.328
**2a**	95.08 ± 2.87	0.84 ± 0.04	0.9980	0.063	0.61 ± 0.01	1.78 ± 0.07	0.9590	0.148
**2b**	137.93 ± 3.63	0.49 ± 0.02	0.9974	0.098	0.79 ± 0.01	0.99 ± 0.04	0.9713	0.651
**3a**	110.86 ± 1.94	2.92 ± 0.11	0.9985	0.077	0.56 ± 0.01	4.32 ± 0.18	0.9626	1.201
**3b**	140.65 ± 3.86	0.45 ± 0.02	0.9984	0.048	0.78 ± 0.01	0.93 ± 0.03	0.9864	0.311

Undoubtedly, adsorption of Fe(III) ions on each hybrid material follows the Langmuir model, which is proven by the calculated *R*^2^ value higher (≥0.997) and χ^2^ values lower (≤0.120) than those for the Freundlich model. This is consistent with the chemical nature of ferric ions, which hinders the intermolecular interactions, leading to the formation of the adsorbate monolayer. On the basis of the Langmuir fitting, the values of maximal adsorption capacity of the materials toward Fe(III) were established. The *q*_max_ values varied between 87.41 and 140.65 mg g^–1^, reaching the highest values for SiO_2_ and Fe_3_O_4_/SiO_2_ particles conjugated with deferoxamine through the maleimide linker. Such a phenomenon might be a result of additional iron ion trapping within a cyclic domain of maleimide. Nevertheless, satisfactory results were obtained for the other materials based on PMVEAMA and silica or SPIONs functionalized through the isocyanate linker. Although the experimental data are not described preferably with the Freundlich model, the values of *1*/*n* constants connected with the intensity of the adsorption process and heterogeneity of the adsorbent’s surface were calculated. The lower the *1*/*n* value, the more intense the adsorption process. For all the materials, the values ranged between 0.56 and 0.79, indicating the efficiency of adsorption processes.

#### 2.3.3 Kinetic Studies of Fe(III) Adsorption

**Figure 6:**
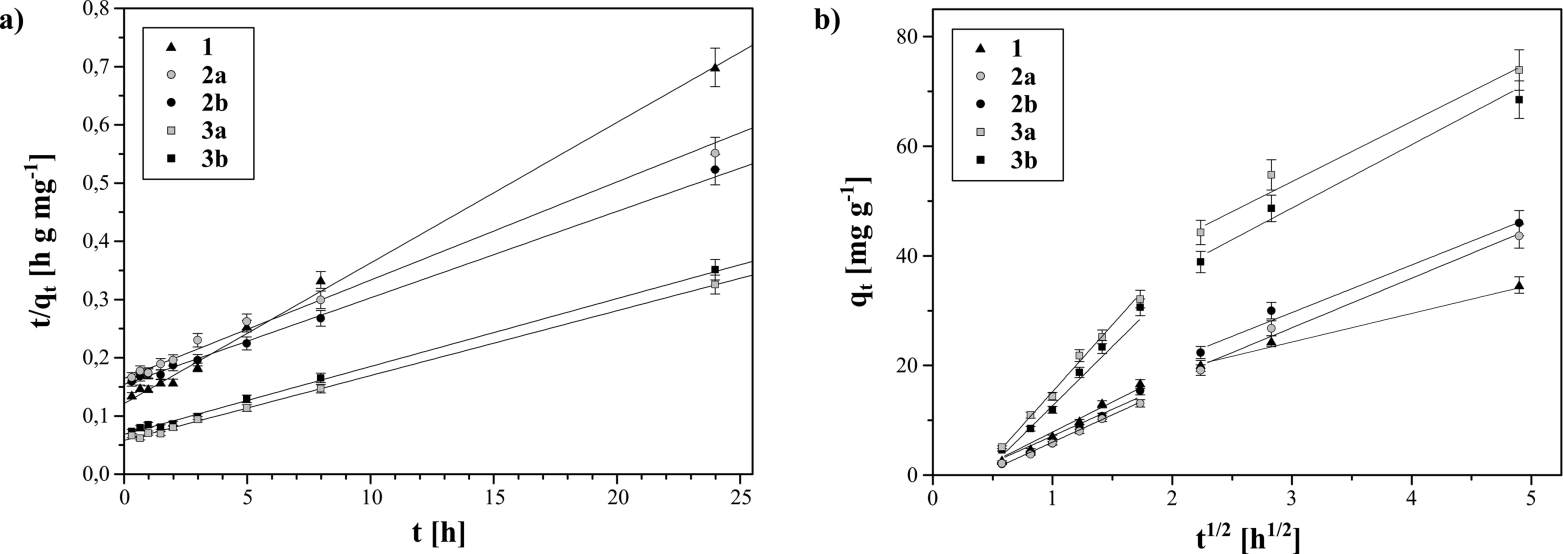
Experimental data fitting to kinetic models: (a) pseudo–second–order; (b) intraparticle diffusion.

The obtained experimental data for the kinetic studies of Fe(III) adsorption on the synthesized hybrid materials were fitted to pseudo–first–order and pseudo–second–order kinetics, intraparticle diffusion theory, and the Elovich model. The highest linear correlation of the experimental data was achieved for the pseudo–second–order kinetic model (Table 3), which plot is presented in Figure 6a. Comparing the calculated *R*^2^ values for the pseudo–second–order kinetic model (Table 3) and the values calculated for the pseudo–first–order kinetic model presented in Figure S7a (Table S1), it is easily shown that the adsorption experiment follows the pseudo–second–order kinetic model. This result implies that the formation of various interactions between the adsorbent and analytes (including electron sharing, the formation of chemical bonds, or proton exchange) is the adsorption rate-limiting step.^31^ Accordingly, the adsorption of Fe(III) ions on the hybrid materials is limited by their coordination by deferoxamine residues on the materials’ surface. The modeling allowed for the calculation of the initial metal ion adsorption rate, which appeared to be the highest for magnetite-based particles and the lowest for silica-based particles. Thus, the time needed for half-adsorption should be opposite to *k_i_* values, which is proven by the lowest *t1/2* values for materials **3a** and **3b**, while the highest for materials **2a** and **2b**. In order to verify the physical nature of the rate-limiting step, the intraparticle diffusion model was implemented to fit the experimental data, in which the plot *q_t_* vs ln *t* may form a multilinear plot, as shown in Figure 6b. The theory introduced by Weber and Morris assumes the multilinearity of the plot when intraparticle diffusion is not the only step limiting the rate of adsorption.^32^ The presented plots show two separate phases; thus, two factors limit the adsorption rate within the adsorption progress. The first one is connected with an initial surface diffusion, while the second one is based on gradual adsorption limited by either intraparticle or pore diffusion. Moreover, the experimental kinetic data were fitted to the Elovich model (Figure S7b) in order to demonstrate whether adsorption of ferric ions on the hybrid materials may include the heterogeneous diffusion process as the rate-limiting step. However, due to the relatively low *R*^2^ values calculated for this model (Table S1), such findings would be deniable.

**Table 3:** Kinetic Parameters Calculated for Pseudo–Second–Order and Intraparticle Diffusion Kinetic Models

	pseudo-second-order				intraparticle diffusion			
					*k*_id_ [mg g^–1^ h^–1/2^]		*R*^2^	
adsorbent	*k*_2_·10^2^ [mg g^–1^ h^–1^]	*k_i_* [mg g^–1^ h^–1^]	*t*_1/2_ [h]	*R*^2^	step 1	step 2	step 1	step 2
**1**	0.48 ± 0.03	8.32 ± 1.26	4.98 ± 0.51	0.9969	12.5 ± 0.9	5.3 ± 0.6	0.9934	0.9941
**2a**	0.17 ± 0.01	6.12 ± 0.96	9.69 ± 0.93	0.9951	9.9 ± 0.4	8.9 ± 1.3	0.9965	0.9906
**2b**	0.14 ± 0.01	6.53 ± 1.07	10.30 ± 1.01	0.9983	11.6 ± 1.7	54.7 ± 9.1	0.9894	0.9251
**3a**	0.22 ± 0.01	17.58 ± 2.32	5.11 ± 0.43	0.9988	23.8 ± 0.3	58.5 ± 0.7	0.9950	0.9995
**3b**	0.20 ± 0.01	14.86 ± 2.04	5.77 ± 0.50	0.9980	23.3 ± 0.4	58.1 ± 0.8	0.9919	0.9977

#### 2.3.4 Thermodynamics of the Adsorption

**Figure 7:**
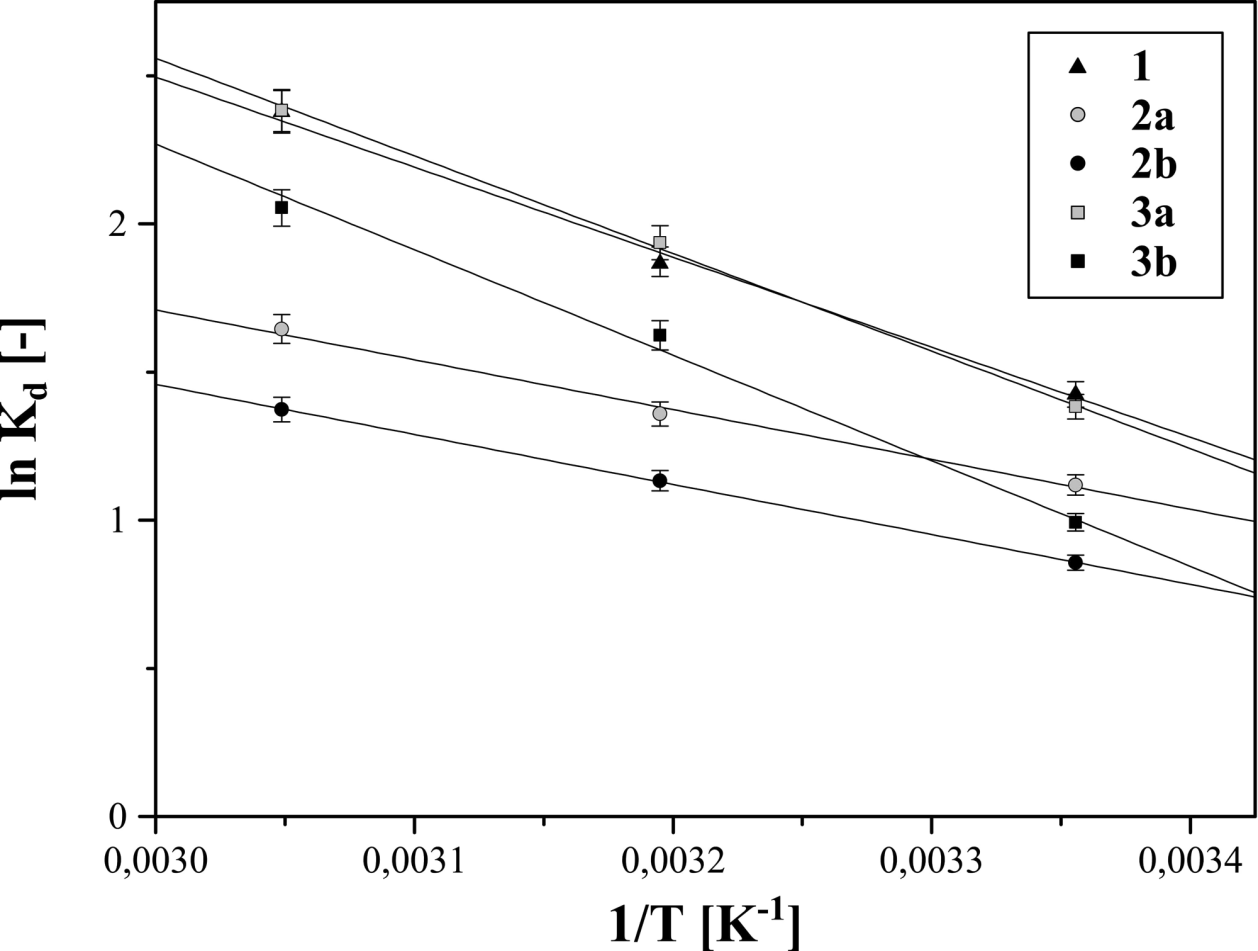
Plots of the van’t Hoff equation fitted to the thermodynamic studies of ferric ion adsorption on the hybrid materials.

Thermodynamic studies involved measurements of the amounts of metal ions absorbed on the deferoxamine-functionalized particles in equilibrium states (after 24 h incubation) in three different temperatures: 298, 313, and 328 K. The obtained experimental data were fitted to the van’t Hoff equation, which linear plots ln*K*_d_ vs *1*/*T* are presented in Figure 7. Based on the calculated slopes and intercepts, three informative parameters were established, i.e., adsorption standard enthalpy (Δ*H*°) and entropy (Δ*S*°) and Gibbs free energy values (Δ*G*°) for experiments conducted under the given thermal conditions. The parameters are collected in Table 4.

**Table 4:** Calculated Thermodynamic Parameters for Fe(III) Adsorption

					Δ*G*° [kJ mol^–1^]		
adsorbent	Δ*H*° [kJ mol^–1^]	Δ*S*° [J mol^–1^ K^–1^]	*R*^2^	χ^2^·(×10^2^)	298 K	313 K	328 K
**1**	25.27 ± 2.14	96.59 ± 6.97	0.9904	0.118	–3.53	–4.86	–6.48
**2a**	13.98 ± 1.57	56.14 ± 5.08	0.9873	0.064	–2.77	–3.54	–4.49
**2b**	14.04 ± 1.26	54.23 ± 4.10	0.9997	0.001	–2.12	–2.95	–3.74
**3a**	27.39 ± 2.13	103.46 ± 6.92	0.9979	0.032	–3.42	–5.04	–6.50
**3b**	29.62 ± 1.71	107.74 ± 5.62	0.9896	0.242	–2.46	–4.23	–5.60

All the plots were characterized with high correlation coefficients *R*^2^, which values were higher than 0.9870, and extremely low χ^2^ coefficients, indicating the good linearity of the plots. Adsorption of Fe(III) ions on the deferoxamine-loaded materials was found to be an endoenergetic process (Δ*H*° values are positive), which is more intense with the temperature increase – values of Gibbs free energies for higher temperature are more negative, and thus, the process is intensified. Interestingly, the established parameters are directly connected with the ones obtained during kinetic studies. The silica-based materials **2a** and **2b** can be described as the least influenced by the temperature increase and the ones that exhibit the lowest adsorption rate constants *k*_2_ and *k_i_* and the highest values of time needed for adsorption of half-equilibrium adsorbate (*t*_1/2_). Moreover, for all the materials, entropy values are relatively high, which suggest that an increased randomness mostly drives the metal ion adsorption at the solution–adsorbent interface related to solvation effects. Such conclusions are rather evident, considering that Fe(III) adsorption is based only on the formation of non-covalent coordination bonds between the receptor and analyte.

#### 2.3.5 Effect of Coexisting Trivalent Ions

The influence of an additional trivalent ion presence on the adsorption selectivity toward ferric ions has been assessed using exemplary silica-based materials **2a** and **2b**. The materials were incubated in three two- or three-component mixtures containing Fe(III), Al(III), and/or Cr(III) ions. The percentages of the metals adsorbed on the hybrid materials were determined using X-ray fluorescence (XRF) measurements (Figure S8) of the material–ion complexes, which are collected in Table 5. Each of the experiments showed that the materials are highly selective toward ferric ions (the percentages of Fe(III) ions are higher than 90%), which is driven by the highest stability constant of the DFO–Fe complex, compared to the complexes with other metal ions. The low, but detectable, contents of Al(III) or Cr(III) might be connected with their chelation by the free deferoxamine residues remaining after the complexation of Fe(III) ions.

**Table 5:** Percentages of the Ions Adsorbed on the Silica-Based Materials **2a** and **2b** Investigated by XRF Analyses

adsorbent	ionic system	percentage of the ions adsorbed [%]		
		Fe	Al	Cr
material **2a**	Fe/Al	93.5	6.5	
	Fe/Cr	91.4		8.6
	Fe/Al/Cr	94.0	2.5	3.5
material **2b**	Fe/Al	97.4	2.6	
	Fe/Cr	95.7		4.3
	Fe/Al/Cr	94.1	2.2	3.7

### 2.4 Chelation of Ferric Ions from the Biological Complex

**Figure 8:**
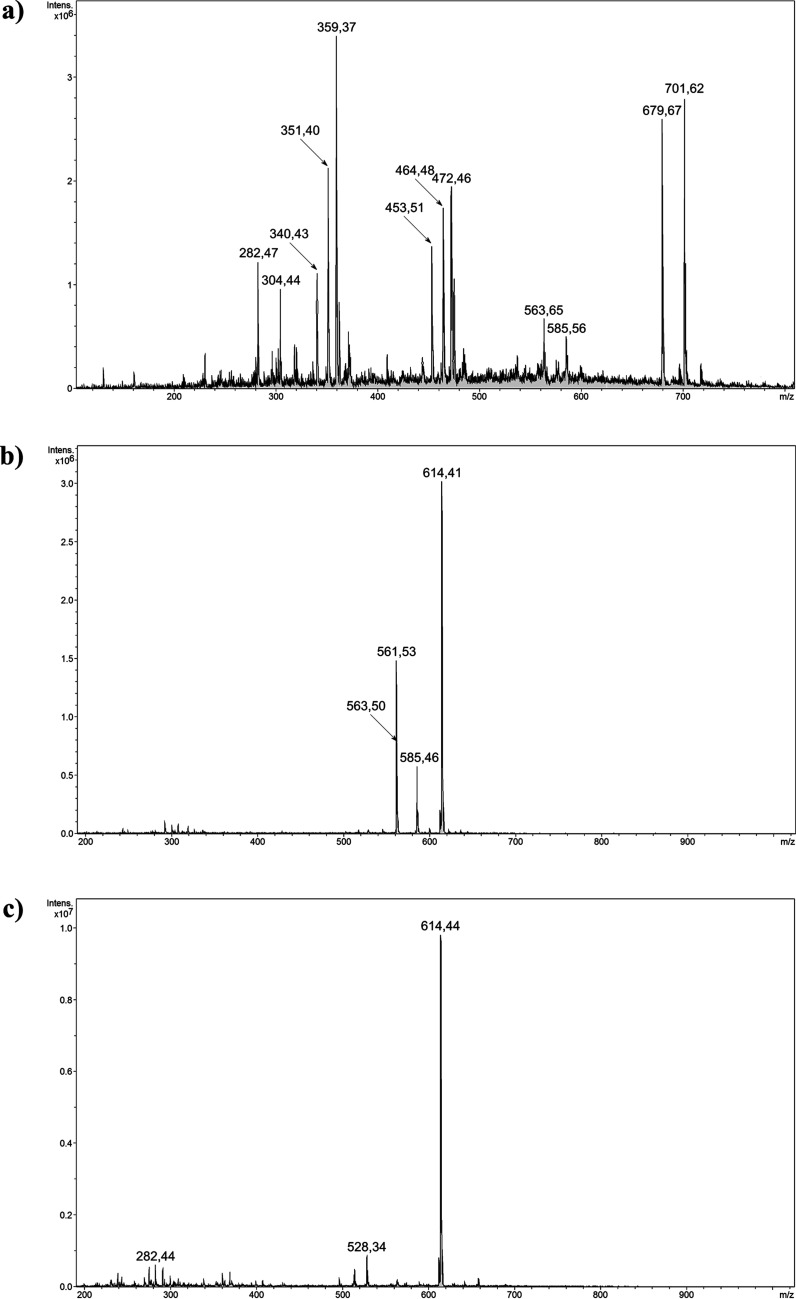
Positive ESI–MS spectra: (a) the formed PPIX–Fe complex; (b) PPIX–Fe complex interaction with pure deferoxamine; (c) PPIX–Fe complex interaction with deferoxamine-loaded Fe_3_O_4_-based hybrid material **3a**.

The synthesized hybrid materials contain biologically compatible platforms and exhibit very promising Fe-adsorptive properties; they can find application in the treatment of diseases caused by long-term or sudden burst release of iron ions in human organisms. The exemplary deferoxamine-loaded hybrid material **3a** was investigated for competitive complexation of Fe(III) ions from the protoporphyrin IX–Fe(III) complex named hemin. PPIX–Fe complex formation and its interaction with Fe_3_O_4_–SiO_2_–NCO–DFO were monitored using electrospray-ionization mass spectrometry (ESI–MS) analysis in positive mode, and the corresponding spectra are presented in Figure 8. The formation of PPIX–Fe complex was undoubtedly proven by signals at 679.7 and 701.6 *m*/*z* appearing in the spectrum in positive mode, which corresponds to the protonated [(PPIX + Fe–2H + 2Na + H_2_O) + H]^+^ form and its sodium adduct, respectively, as well as their bicharged forms at 340.4 and 351.4 *m*/*z*. Also, the signal at 359.4 *m*/*z* is related to the bicharged protonated sodium adduct of the [(PPIX + Fe + Cl–2H + 2Na) + H + Na]^2+^ form. The similar trend of the signals distances as for 340.4, 351.4, and 359.4 *m*/*z* is visible for the signals at 453.5, 464.5, and 472.5 *m*/*z,* indicating PPIX–Fe complex adducts. Although the spectrum exhibits mono- and bicharged signals corresponding to free porphyrin domains not complexing Fe(III) ions (*m*/*z* 282.5 [PPIX + 2H]^2+^; 304.4 [PPIX + 2Na]^2+^; 563.6 [PPIX + H]^+^; 585.6 [PPIX + Na]^+^), their intensity is relatively low, which proves high Fe(III)-complexing efficiency. The obtained PPIX–Fe complex treated with an excess of deferoxamine led to a complete transfer of Fe(III) ions to the DFO domain, which is proven by both the appearance of a signal at 614.4 *m*/*z* [DFO–2H + Fe]^+^ and the disappearance of signals corresponding to the PPIX + Fe complex in Figure 8b. Moreover, the spectrum shows two signals related to free PPIX at 563.5 and 585.5 *m*/*z* and a signal of free deferoxamine at 561.5 *m*/*z* caused by its excessive usage in the experiment, which proves the competitive extraction of iron ions by the studied siderophore. These results have prompted examining the hybrid materials’ potential for competitive chelation of ferric ions, which was conducted using material **3a** as an exemplary scavenger. After a very short incubation of the material in the PPIX–Fe complex solution, the aqueous phase was analyzed, the spectrum of which is given in Figure 8c. The most intensive signal at 614.4 *m*/*z* corresponds to DFO–Fe complex, which is a consequence of a nanosized character of the material used, not fully separable within the short time of magnetic separation, and thus getting to the ionization source. Nevertheless, it confirmed a very efficient competitive binding of Fe(III) ions within the deferoxamine-loaded material’s matrix, leaving PPIX uncomplexed. Similar results were observed for the samples containing material **3a** incubated in a series of buffer solutions, which were citric acid/sodium hydrogen phosphate buffers of pH values ranging between 3 and 8, and phosphate-buffered saline (PBS) of pH 7.4. The choice of such conditions was triggered by the different pH of fluids in human organisms. The corresponding ESI–MS spectra presented the signals originating from the ingredients of buffers and the signal referring to the complex of DFO and Fe(III) ions, as a pending organic domain on the not fully separated adsorptive nanoparticles. Therefore, the exemplary material **3a** was proven for efficient ferric ion transfer from its PPIX complex in a wide range of aqueous environments.

Very satisfactory results of direct or competitive complexing of ferric ions may lead to the materials’ further biological applications, especially the materials based on magnetically active Fe_3_O_4_ nanoparticles, which have already been proven as valuable targeted drug- or energy-transporting platforms. One of the reasons for their high *in vitro* and/or *in vivo* applicability is their nanosize allowing for an enhanced circulation within the bloodstream. Also, the paramagnetic character of Fe_3_O_4_ nanoparticles allows for targeted transport based on the three-dimensional concentration of the particles using an external point or rotating magnetic field.^33−36^ Not only such features lead to a directed transport of drugs to target sites (such as tumors, organs, tissues, etc.), improving the selectivity of the therapeutic effect, but also Fe_3_O_4_-based systems exhibit efficiency in hyperthermia treatment, thanks to the possibility of heat generation at the specific organism site.^37^ The paramagnetic features of magnetite-based materials functionalized with deferoxamine were examined using materials **3a** and **3b**. Figure S9 presents the concentration of the particles using a neodymium magnet in two different media (human serum and prepared phosphate-buffered saline), which afford physiological or paraphysiological conditions. The particles are easily concentrated with the magnet even at a distance between the magnet and the sample of 5 cm, which indicates the easy directing of the particles using the external magnetic field. Therefore, the magnetite-based materials can find application as iron ion scavengers at targeted organism sites.

## 3 Conclusions

The recent study presents the synthesis of deferoxamine-loaded hybrid materials using three different supports. The obtained materials were characterized with several analytical techniques, involving the characterization of raw hybrid materials and their complexes with Fe(III) ions. The materials were subjected to various adsorption studies, which responded in the comprehensive characterization of the materials’ adsorptive potential toward ferric ions. All the materials showed high effectiveness in the adsorbate binding, reaching adsorption capacities of 87.41 to 140.65 mg g^–1^, which correspond to 1.56 to 2.52 mmol g^–1^. The highest values were obtained for magnetite-based materials **3a** and **3b**, which might be related to the use of a nanosized support. Moreover, material **3a**, as a magnetically active exemplary material, was studied for competitive binding of Fe(III) ions from their complex with PPIX, corresponding to hemin – an iron-containing porphyrin found in human blood. The material exhibited high effectiveness, which, jointly with its ability for being magnetically directed, may lead to further biological application in the treatment of hematologic diseases.

## 4 Materials and Methods

### 4.1 Chemicals

The majority of the reagents were obtained from Sigma Aldrich (Saint Louis, USA) (deferoxamine mesylate salt ≥92.5%; poly(methyl vinyl ether-*alt*-maleic anhydride) of average *M*_w_ ∼216,000 Da and *M*_n_ ∼80,000 Da; tetraethyl orthosilicate ≥99.0%, 3-(triethoxysilyl)propyl isocyanate 95%; (3-aminopropyl)triethoxysilane 97%; maleic anhydride 99%; 1,1,1,3,3,3-hexamethyldisilazane for synthesis ≥98%; ZnCl_2_ ≥98%, anhydrous; Fe(ClO_4_)_3_·*x*H_2_O, low chloride; protoporphyrin IX (PPIX) ≥95%; human serum from human male AB plasma, USA origin, sterile-filtered). Silica modified with surface isocyanate and maleimide groups was obtained from SiliCycle Inc. (Quebec, Canada) and characterized as follows: SiO_2_–maleimide (size: 40–63 μm, loading: 0.68 mmol g^–1^) and SiO_2_–isocyanate (size: 40–63 μm, loading: 1.41 mmol g^–1^). The other substances were of purity grade p.a. and obtained from POCH (Gliwice, Poland) (FeCl_3_·6H_2_O ≥97%; Na_2_HPO_4_·2H_2_O ≥99%; NaH_2_PO_4_·H_2_O ≥99%; Et_2_O 99.5%; KCl 99.5%; anhydrous EtOH 99.8%; NH_4_OH 25%), Stanlab (Lublin, Poland) (HCl 35–38%; citric acid monohydrate; toluene), and EUROCHEM (Tarnów, Poland) (NaCl 99.5%; DMF, DCM). Moreover, (NH_4_)_2_Fe(SO_4_)_2_·6H_2_O was supplied by Aktyn (Suchy Las, Poland), and DMSO was purchased from Merck (Darmstadt, Germany), which was dried over molecular sieves 4 Å prior to its use.

### 4.2 Instruments

Characterization of materials involved using several analytical techniques. The FT-IR spectra of deferoxamine-loaded hybrid materials were recorded on a Bruker IFS 66v/S (Bremen, Germany) spectroscope operating in the wavelength range between 400 and 4000 cm^–1^ with a resolution of 2 cm^–1^, using KBr pellets as the sample medium. The thermogravimetric measurements were performed using a Setaram Setsys 1200 analyzer (Caluire, France) operating between 20 and 1000 °C with a heating rate set for 5 °C min^–1^. The samples were heated in an airstream. The quantities of C, H, and N contents in the hybrid materials were calculated based on the elemental analysis performed in an Elementar Vario EL III analyzer (Langenselbold, Germany). The obtained DFO-loaded materials were also visualized using an FEI Quanta FEG 250 (Hillsboro, OR, USA) scanning electron microscope (SEM) operating in a high vacuum condition of 70 Pa. The instrument used an accelerating voltage of 10 kV and a working distance varying between 9.9 and 10.4 mm. The SEM images were obtained with magnitudes of 2000× for materials **1**, **2a**, and **2b** and of 100,000× for Fe_3_O_4_-based materials **3a** and **3b**. Moreover, for non-magnetite-based materials, energy dispersive X-ray spectroscopic (EDX) imaging was performed. The magnetite, magnetite–silica, and magnetite-based hybrid materials were also characterized using a Bruker D8 Advance (Bremen, Germany) powder diffractometer (XRD). The apparatus used Cu Kα1 X-ray energy of wavelength λ of 1.5406 Å (Johansson type) and worked in a high-angle mode in the 2θ range between 6 and 60°. Also, magnetite-based materials **3a** and **3b** and the exemplary complex of material **3a** with adsorbed Fe(III) ions were characterized with the Brunauer–Emmet–Teller (BET) isotherm performed using a Quantachrome Autosorb iQ (Boynton Beach, USA). The samples were outgassed for 12 h at 100 °C prior to their analysis for nitrogen adsorption and desorption at a temperature of 77.35 K. According to the experimental data, the samples’ surface areas were established using the BET and Barrett–Joyner–Halenda (BJH) methods. The range of relative pressure *p*/*p_0_* used for the measurements was from 0.0 to 1.0, where the applicability of the methods is from 0.05 to 0.3 and from 0.1 to 1.0 for the BET and BJH methods, respectively.

The progress of Fe(III) adsorption on the DFO-loaded hybrid materials was monitored by UV–Vis assays using an Agilent 8453 spectrophotometer (Santa Clara, USA), operating in the range of wavelengths between 200 and 1000 cm^–1^ with a resolution of 1 cm^–1^. The samples were placed in a poly(methyl methacrylate) (PMMA) cuvette (optical path length: 10 mm), and the spectra were recorded in triplicate in order to avoid any disturbances. The competitiveness in trivalent ion binding by materials **2a** and **2b** was monitored using a MiniPal2 X-ray spectrofluorometer (XRF) supplied by Malvern PANalytical B.V. (Almelo, Netherlands) equipped with a rhodium vacuum tube as a source of X-rays. The analyses were performed for 200 s with an X-ray tube voltage of 13 kV and automatically adopted current, which varied between 18 and 30 μA. Moreover, the measurements were conducted using a no element-excluding filter.

The materials’ stability and the competitive binding of Fe(III) ions by the chosen material **3a** from a prepared PPIX–Fe (hemin) complex were monitored using an amaZon SL ion trap Bruker (Bremen, Germany) mass spectrometer with an electrospray ionization source (ESI–MS). The samples were injected into the ionization source at a flow rate of 10 μL min^–1^ by a syringe pump. The spectrometer worked in a so-called “enhanced resolution mode” and a detection range between 100 and 1000 *m*/*z*. The desolvating gas (N_2_) flowed at a rate of 800 L h^–1^, while the cone gas (He) flowed at a rate of 50 L h^–1^. The voltages were set at −4.5 and −0.5 kV for the capillary and the endplate offset, respectively.

### 4.3 Synthesis of the Deferoxamine-unctionalized Hybrid Materials

#### 4.3.1 PMVEAMA-Based Material

A solution of deferoxamine mesylate (0.84 g; 1.28 mmol) in 30 mL of DMF was placed in a three-neck round-bottom flask and charged with a reflux condenser. The solution was purged with inert gas (N_2_) and heated to 110 °C. Under a nitrogen atmosphere, a solution of PMVEAMA (0.4 g) in 30 mL of toluene:DMF mixture (2:1; v:v) was added dropwise through a dropping funnel, with subsequent heating and mixing for 16 h. Afterward, a cooled solution was treated with Et_2_O to obtain the brown precipitate. The crude product was dissolved in EtOH and recrystallized with Et_2_O, yielding 1.16 g (93.5%) of material **1**.

Material **1**: FT-IR (KBr) cm^–1^: 3399 (broad, ν O–H, N–H stretch), 2930 (ν C–H asym stretch), 2859 (ν C–H sym stretch), 1769 (ν C=O anhydride ring stretch), 1700 (ν C=O carboxylic acid stretch), 1654 (ν C=O amide stretch), 1558 (ν N–H amide bend), 1438 (ν O–H bend), 1193 (ν C–O stretch), 1051 (ν N–OH stretch), 785 (ν N–H bend).

#### 4.3.2 Silica-Based Hybrid Materials

The anchoring of deferoxamine on the silica modified with either isocyanate or maleimide groups was based on the same synthetic protocol. A solution of deferoxamine mesylate (0.52 g; 0.8 mmol) in 40 mL of DMF was heated to ∼75 °C and purged with nitrogen. Then, 2 g of isocyanate- or maleimide-modified was added in a few portions. Mixing under an inert atmosphere at elevated temperature was continued for 2 h for isocyanate-functionalized silica particles or 5 h for maleimide-functionalized silica particles, leading to material **2a** or material **2b**, respectively. Afterward, the warm mixture was filtered off, and the solid was washed with DMF (30 mL) and DCM (25 mL), obtaining white material **2a** and yellow material **2b**.

Material **2a**: FT-IR (KBr) cm^–1^: 3427 (broad, ν O–H, N–H stretch), 2939 (ν C–H asym stretch), 2891 (ν C–H sym stretch), 1647 (ν C=O amide stretch), 1572 (ν N–H amide bend), 1092 (broad, ν Si–O–Si sym stretch), 805 (ν Si–O–Si asym stretch), 467 (ν Si–O–Si bend).

Material **2b**: FT-IR (KBr) cm^–1^: 3447 (broad, ν O–H, N–H stretch), 2926 (ν C–H asym stretch), 2854 (ν C–H sym stretch), 1709 (ν C=C maleimide stretch), 1655 (ν C=O amide stretch), 1414 (ν O–H bend), 1094 (broad, ν Si–O–Si sym stretch), 958 (ν Si–OH bend), 803 (ν Si–O–Si asym stretch), 698 (ν = C–H maleimide bend), 467 (ν Si–O–Si bend).

#### 4.3.3 Magnetite-Based Hybrid Materials

##### 4.3.3.1 Synthesis of the Fe_3_O_4_–SiO_2_ Platform

Magnetic iron oxide (II,III) was obtained using the standard coprecipitation method. An aqueous solution of FeCl_3_·6H_2_O (10.81 g; 0.04 mol) and (NH_4_)_2_Fe(SO_4_)_2_·6H_2_O (7.84 g; 0.02 mol) in 400 mL of distilled water in a three-neck round-bottom flask was purged with N_2_. During mixing and constant purging with the inert gas, a solution of 30 mL of NH_4_OH in 20 mL of distilled water was added dropwise with immediate precipitation of magnetite. After ammonia was added, the mixture was stirred for 1 h. The precipitate was collected using an external neodymium magnet, then washed two times with distilled water (50 mL) and two times with ethanol (50 mL), and dried under vacuum at 50 °C for 8 h, yielding magnetic nanoparticles (4.52 g; 97.6%). The obtained Fe_3_O_4_ nanoparticles were further encapsulated within the silica matrix using the standard Stöber method. The dried magnetite nanoparticles were dispersed in 300 mL of H_2_O:EtOH mixture (2:1; v:v) on an ultrasound bath at room temperature. Next, 30 mL of ammonia was added. During continuous stirring, a solution of TEOS (490 μL; 2.25 mmol) in 20 mL of EtOH was added dropwise. The mixture stayed in the ultrasound bath for 3 h, obtaining dark brown particles. Afterward, the solid was separated using the magnet, washed two times with distilled water (50 mL) and two times with ethanol (50 mL), and dried under vacuum at 50 °C for 8 h, yielding Fe_3_O_4_–SiO_2_ platform.

##### 4.3.3.2 Synthesis of Fe_3_O_4_–SiO_2_–NCO–Deferoxamine

To a solution of deferoxamine mesylate (0.79 g; 1.2 mmol) in 50 mL of anhydrous DMSO placed in the ultrasound bath, (3-isocyanatopropyl)triethoxysilane (297 μL; 1.2 mmol) was added. After mixture stirring for 2 h under an inert gas atmosphere, 2.4 g of Fe_3_O_4_–SiO_2_ particles was added in a few portions. The silyl-derivative incorporation into the silica matrix covering the magnetite core was carried out for 16 h at room temperature under an inert gas atmosphere (N_2_). The resulting deferoxamine-modified magnetite-based hybrid material was separated with a magnet, washed two times with EtOH (20 mL) and two times with DCM (20 mL), and then dried under vacuum at 50 °C for 8 h. The resulting dark brown particles were assigned as material **3a**.

Material **3a**: FT-IR (KBr) cm^–1^: 3384 (broad, ν O–H, N–H stretch), 2918 (ν C–H asym stretch), 2855 (ν C–H sym stretch), 1634 (ν C=O amide stretch), 1435 (ν O–H bend), 1018 (ν Si–O–Si sym stretch), 953 (ν Si–OH bend), 795 (ν Si–O–Si asym stretch), 582 (ν Fe–O stretch).

##### 4.3.3.3 Synthesis of Fe_3_O_4_–SiO_2_–Maleimide–Deferoxamine

Synthesis of deferoxamine-functionalized magnetic particles through the maleimide linker was based on a synthesis of 3-maleimide-propyltriethoxysilane, with its further anchoring to Fe_3_O_4_–SiO_2_ surface, which then underwent functionalization with deferoxamine. The maleimide-derivative was obtained in a three-step process: (1) To a solution of maleic anhydride (0.35 g; 3.6 mmol) in 60 mL of anhydrous DMSO, 3-aminopropyltriethoxysilane (842 μL; 3.6 mmol) was added. The ring-opening process was handled under continuous stirring for 2 h at room temperature. (2) Then, the mixture was heated to 80 °C in an oil bath, and a solution of hexamethyldisilazane (755 μL; 3.6 mmol) in a DMSO:toluene mixture (2:1; v:v) and ZnCl_2_ (0.49 g; 3.6 mmol) were added. The reaction mixture was further stirred for 5 h at 80 °C. (3) Afterward, the mixture was cooled to room temperature, and then the unreacted reagents were extracted with cold Et_2_O (3 × 75 mL). The obtained DMSO solution of the silane maleimide-derivative was poured to Fe_3_O_4_–SiO_2_ (2.4 g) suspension dispersed in 40 mL of anhydrous DMSO. The silane binding to the surface silica matrix was continued for 16 h under a nitrogen atmosphere at room temperature. The obtained Fe_3_O_4_–SiO_2_–maleimide particles were separated, washed one time with DMSO and three times with EtOH, and then dried under vacuum at 50 °C. The dried particles were then poured into a solution of deferoxamine mesylate (0.79 g; 1.2 mmol) in 50 mL of anhydrous DMSO. The reaction of deferoxamine functionalization in the ultrasound bath was carried out for 24 h at room temperature, under a nitrogen atmosphere. The resulting dark brown particles were separated, washed with fresh solvents (1 × 10 mL DMSO, 3 × 20 mL EtOH), and dried under vacuum (50 °C), obtaining material **3b**.

Material **3b**: FT-IR (KBr) cm^–1^: 3396 (broad, ν O–H, N–H stretch), 2930 (ν C–H asym stretch), 2855 (ν C–H sym stretch), 1700 (ν C=C maleimide stretch), 1630 (ν C=O amide stretch), 1577 (ν N–H amide bend), 1404 (ν O–H bend), 1050 (ν Si–O–Si sym stretch), 797 (ν Si–O–Si asym stretch), 582 (ν Fe–O stretch).

### 4.4 Stability of Deferoxamine-Functionalized Materials

The materials’ stability in biological conditions was investigated by incubation of 15 mg samples of each material in 20 mL of preprepared phosphate-buffered saline (PBS), which affords paraphysiological conditions. The incubation was handled at 37 °C for 24 h with constant shaking. Afterward, the solids were centrifuged, and the solutes were injected for ESI–MS analysis.

### 4.5 Fe(III) Adsorption Experiments

#### 4.5.1 The Influence of pH on Adsorption Processes

Adsorption processes in different aqueous environments were performed using 5 mM solutions of Fe(ClO_4_)_3_·6H_2_O buffered in prepared solutions of pH 1 and 2 (hydrochloric acid/potassium chloride buffer), pH 3, 4, and 5 (citric acid/disodium hydrogen phosphate), and pure distilled water. Each experiment involved using 10 mg of sample of each hybrid material, which was poured into 10 mL of Fe(III) ion solution buffered in a particular medium. Each sample was shaken for 24 h at room temperature. Afterward, the solids were separated by filtration using a Schott funnel, centrifugation, or using a magnet, depending on the type of material used. The solutions were then investigated for the amount of remaining metal ions, established using UV–Vis spectrophotometric measurements (λ_max_ = 297 nm). The amount of Fe(III) absorbed *q*_eq_ on the hybrid material was calculated using the below equation, where *c*_0_ and *c*_eq_ are the initial and the equilibrium concentrations of the metal used, respectively [mM], *V* is the volume of the solution used [mL], *m* is the sample mass [mg], and *M* is the molar mass of the adsorbate [g mol^–1^].
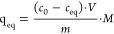


#### 4.5.2 Adsorption Isotherms

Isothermal studies involved adsorption of Fe(III) ions from their perchlorate salt aqueous solution in distilled water at different concentrations: 0.1, 0.5, 1, 2, 5, 10, and 20 mM. The experimental protocol and the quantification of the metal adsorbed on the materials were similar to those described in Section 4.5.1. The obtained experimental data were then fitted to two widely used isothermal models: the Langmuir and the Freundlich models, which are presented below, respectively
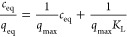

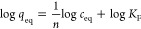
where *q*_max_ is the maximal adsorption capacity of the material toward the studied analyte [mg g^–1^], *K*_L_ is the Langmuir adsorption constant related to the analyte affinity to the adsorption binding sites [L mg^–1^], 1/*n* is the empirical constant indicating the heterogeneity of the adsorbent, and *K*_F_ is the Freundlich adsorption constant characteristic at a given temperature [mg g^–1^ (L mg^–1^)^1/*n*^].

#### 4.5.3 Adsorption Kinetics

The performing of kinetic studies was based on the quantification of the metal uptake from its aqueous solution depending on the contact time. Thus, 15 mg of sample of each hybrid material was added to 20 mL of 5 mM solution of Fe(III) ions in distilled water. The solute was collected in preset time intervals (0.25, 0.5, 0.75, 1, 2, 3, 5, 8, and 24 h), in order to calculate the collective amount of metal absorbed *q_t_* at time *t*, using the below equation (analogical to the equation adopted to the equilibrium state), where *c_t_* is the concentration of the metal at time *t* [mM]:
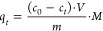
The obtained experimental data were subsequently fitted to several kinetic models, leading to the decent characterization of the adsorption processes. The models used were the pseudo–first–order kinetic model, the pseudo–second–order kinetic model, the intraparticle diffusion model, and the Elovich model, in which linear plots are given below in appropriate order
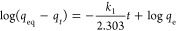

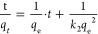

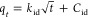

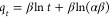
where *q*_e_ is the calculated equilibrium amount of metal absorbed [mg g^–1^], *k*_1_ is the pseudo-first-order kinetics constant [h^–1^], *k*_2_ is the pseudo-second-order kinetics constant [g mg^–1^ h^–1^], *k*_id_ is the intraparticle diffusion constant [mg g^–1^ h^–1/2^], *C*_id_ is the intraparticle diffusion plot intercept [mg g^–1^], α is the Elovich constant [mg g^–1^ min^–1^], and β is the Elovich exponent [mg g^–1^]. Moreover, based on the pseudo-first- and pseudo-second-order kinetic fitting, initial adsorption rate constants *k_i_* [mg g^–1^ h^–1^] were calculated, respectively
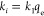

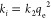
Linear fitting of the experimental data to the pseudo–second–order kinetic model also allowed for establishing the half-adsorption time *t*_1/2_ [h], which is equal to the time needed for adsorption of the half amount of analyte adsorbed in equilibrium:
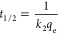


#### 4.5.4 Adsorption Thermodynamics

Thermodynamic studies were based on the reaching Fe(III) adsorption equilibrium state at three different incubation temperatures: 298, 313, and 328 K. To 5 mL of ferric perchlorate aqueous solution in distilled water of concentration 5 mM, 10 mg of sample of the hybrid material was introduced. Each mixture was incubated in 298, 313, or 328 K for 24 h. Afterward, the amount of metal adsorbed on the hybrid material was calculated using the same protocol as given in Section 4.5.1. The obtained data were fitted to the van’t Hoff equation, which is given below
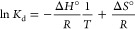
where *K*_d_ is the distribution coefficient [L g^–1^] calculated as *q*_eq_/*c*_eq_, *R* is the ideal gas constant (8.314 J mol^–1^ K^–1^), and Δ*H*° and Δ*S*° are the standard enthalpy [J mol^–1^] and entropy [J mol^–1^ K^–1^], respectively, of Fe(III) adsorption on the particular hybrid material. Moreover, values of Gibbs free energies Δ*G*° [J mol^–1^ K^–1^] in particular conditions were calculated:
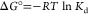


### 4.6 Competitive Binding of Trivalent Ions

A series of 50 mg samples of silica-based materials **2a** and **2b** were incubated for 24 h with 10 mL samples of three different Fe-containing mixtures of trivalent ions (Fe/Cr/Al, Fe/Cr, or Fe/Al systems). The mixtures, which contained Fe(ClO_4_)_3_, Cr(ClO_4_)_3_, and/or Al(ClO_4_)_3_ at their final concentration of 5 mM, were prepared using distilled water as a solvent. After the incubation time, the solids were centrifuged, the solutes were separated, and the material–ion complexes were dried under vacuum in the desiccator at room temperature. The dried samples were subjected to XRF analysis.

### 4.7 Chelation of Ferric Ions from the Biological Complex

In order to investigate the competitiveness of the materials in binding Fe(III) ions, the exemplary material **3a** was studied using a complex of protoporphyrin IX (PPIX) and Fe(III) ions. The complex was obtained by mixing 5 mL of a 0.1 mM solution of PPIX in methanol with 5 mL of a 0.1 mM solution of Fe(III) ions in distilled water for 24 h at room temperature. Afterward, 2.5 mL of the obtained PPIX–Fe complex was diluted with 2.5 mL of H_2_O:MeOH (1:1) mixture, leading to the final complex concentration of 0.025 mM, and 10 mg of **3a** was purged into the solution. The mixture was shaken for 1 min, and then the material was separated using an external magnetic field. The formation of PPIX–Fe and the progress of Fe-binding within the adsorptive material were monitored using ESI–MS analysis. Moreover, the competitiveness studies were also performed using pre-prepared citric acid/sodium hydrogen phosphate buffers of pH 3, 4, 5, 6, 7, and 8 and phosphate-buffered saline (PBS). Briefly, 0.5 mL of a 0.5 mM solution of PPIX–Fe in a mixture of H_2_O:MeOH (1:1) was added to 9.5 mL of the buffers, and then 10 mg samples of **3a** were added. The incubation and analysis conditions were the same as described above.

## Supporting Information Available

A presentation of the synthetic protocols for obtaining the DFO-loaded hybrid materials; the XRD spectra of magnetite-based materials; the BET isotherms and the pore size distribution profiles of Fe_3_O_4_-cored materials **3a**, **3a–Fe(III)**, and **3b**; the ESI–MS spectra of free deferoxamine and its complexes with Fe(III) and Fe(II) ions; additional plots and coefficients calculated for the adsorption experiments; the XRF spectra of materials **2a** and **2b** complexed with other co-existing trivalent ions; the images of the magnetite-based materials concentrated using a neodymium magnet (PDF)
