# Supplementary material for: Deferoxamine-Modified Hybrid Materials for Direct Chelation of Fe(III) Ions from Aqueous Solutions and Indication of the Competitiveness of In Vitro Complexing toward a Biological System
Source: ACS Omega. 2021 Jun 3;6(23):15168–81. doi: 10.1021/acsomega.1c01411 (PMC8210399; doi:10.1021/acsomega.1c01411)
Supplement: Supplementary file 1 — ao1c01411_si_001.pdf [file ao1c01411_si_001.pdf]

Supporting Information

Deferoxamine–Modified Hybrid Materials for Direct Chelation of Fe(III) Ions from Aqueous Solutions and Indication of the Competitiveness of *In Vitro* Complexing toward a Biological System

Mateusz Pawlaczyk*, Grzegorz Schroeder

Faculty of Chemistry, Adam Mickiewicz University in Poznań, Uniwersytetu Poznańskiego 8, 61–614 Poznań, Poland

* Corresponding author: Mateusz Pawlaczyk, mateusz.pawlaczyk@amu.edu.pl, tel: +48 61 829 17 97

Figure captions:

Figure S1. Synthetic routes for obtaining the deferoxamine–functionalized hybrid materials

Figure S2. Top: XRD spectra of the synthesized bare Fe_3O_4 and Fe_3O_4–SiO_2–NCO–DEF (**3a**) and Fe_3O_4–SiO_2–maleimide–deferoxamine (**3b**) hybrid materials; Bottom: a representation of theoretical XRD pattern of Fe_3O_4 (blue) and SiO_2 (red) (JCPDS card no. 01–088–0315)

Figure S3. BET isotherms obtained for material **3a** before and after the complexation of Fe(III) ions, and for material **3b**. The red line refers to the nitrogen adsorption, while the blue line to the desorption step

Figure S4. The pore size distribution of materials **3a** (green), **3b** (blue), and the complex material **3a**–Fe(III) (red)

Figure S5. The ESI–MS spectra in positive mode of aqueous solutions: a) pure deferoxamine mesylate; b) deferoxamine–Fe(III) complex using $FeCl_3$; c) deferoxamine–Fe(II) complex using Mohr's salt ($(NH_4)_2Fe(SO_4)_2$)

Figure S6. The experimental data fitting to the Freundlich isothermal model

Figure S7. The kinetic experimental data fitted to: a) the pseudo–first–order kinetic model; b) the Elovich model

Figure S8. The XRF spectra of the complexes of materials **2a** and **2b** with trivalent metal cations. The materials were incubated with three different systems, containing Fe(III), Al(III), and/or Cr(III) cations

Figure S9. Concentrating of material **3a** (a, b) and **3b** (c, d) by neodymium magnet in: PBS buffer (a, c) and human serum (b, d). The distances between magnet and sample are 0 cm (left) and 5 cm (right)

Table heading:

Table S1. Additional kinetic parameters calculated for Fe(III) adsorption kinetics fitted to the pseudo–first–order and the Elovich models

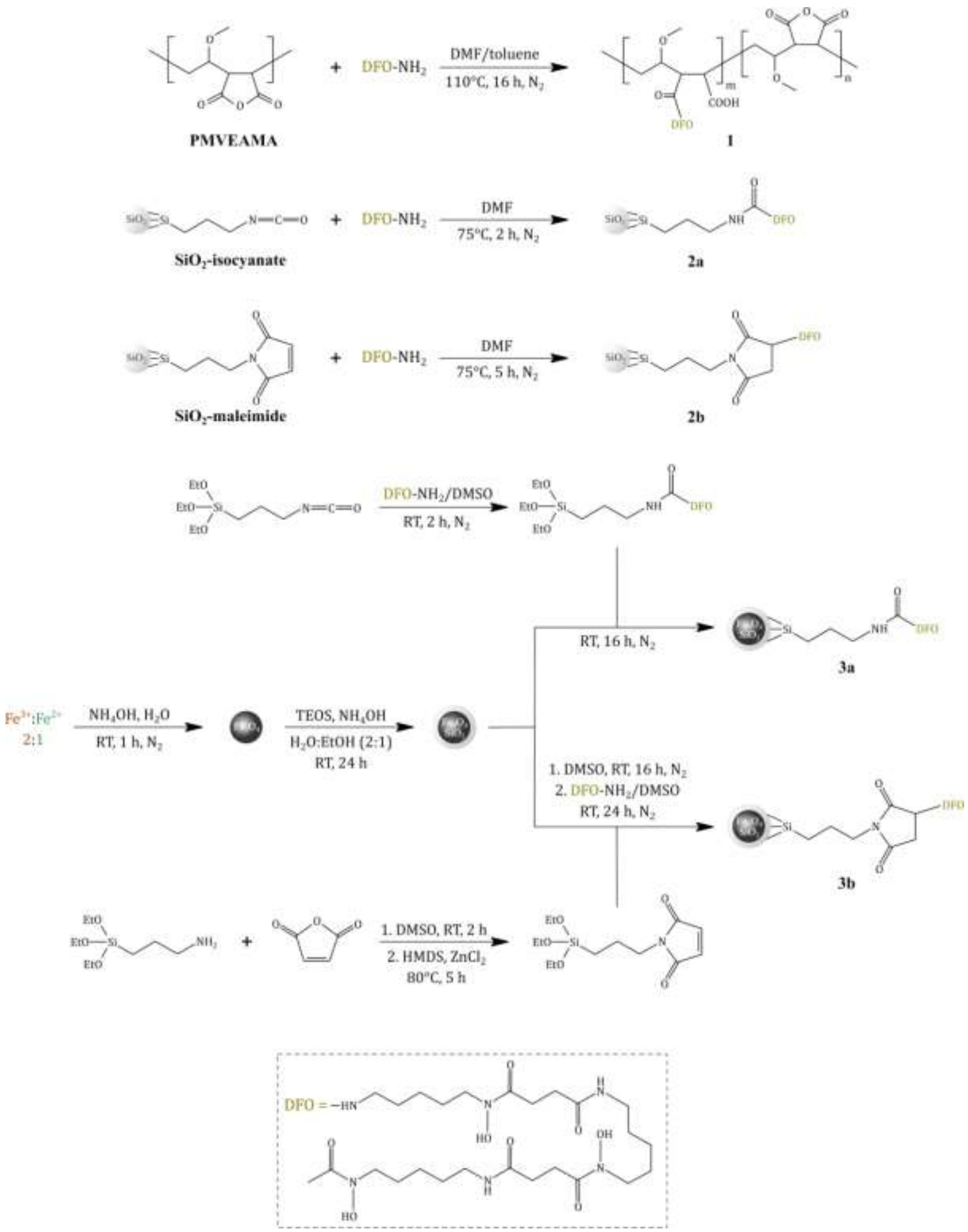

Figure S1. Synthetic routes for obtaining the deferoxamine–functionalized hybrid materials

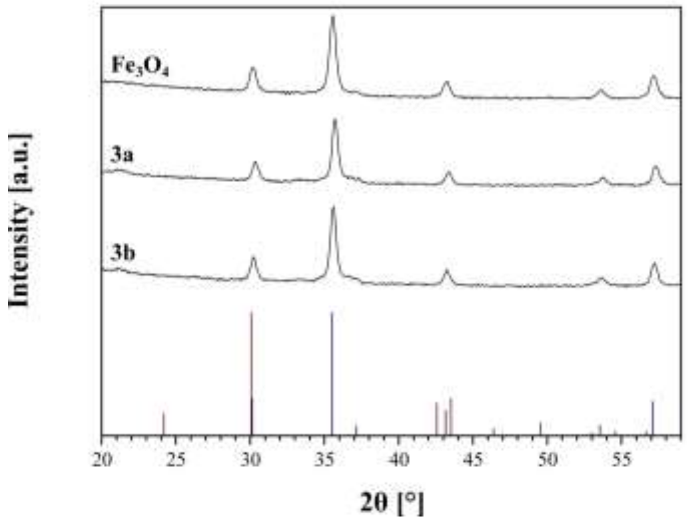

Figure S2. Top: XRD spectra of the synthesized bare Fe_3O_4 and Fe_3O_4–SiO_2–NCO–DEF (**3a**) and Fe_3O_4–SiO_2–maleimide–deferoxamine (**3b**) hybrid materials; Bottom: a representation of theoretical XRD pattern of Fe_3O_4 (blue) and SiO_2 (red) (JCPDS card no. 01–088–0315)

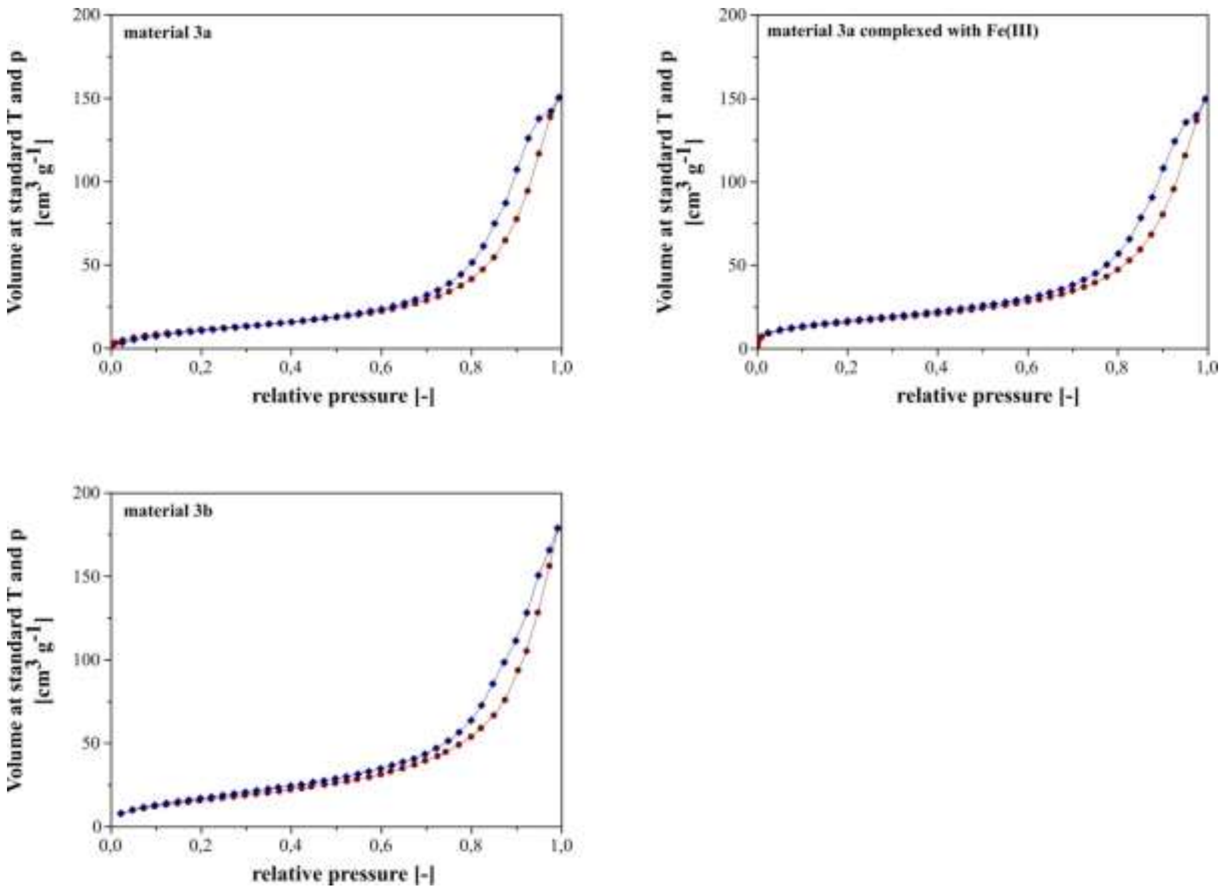

Figure S3. BET isotherms obtained for material **3a** before and after the complexation of Fe(III) ions, and for material **3b**. The red line refers to the nitrogen adsorption, while the blue line to the desorption step

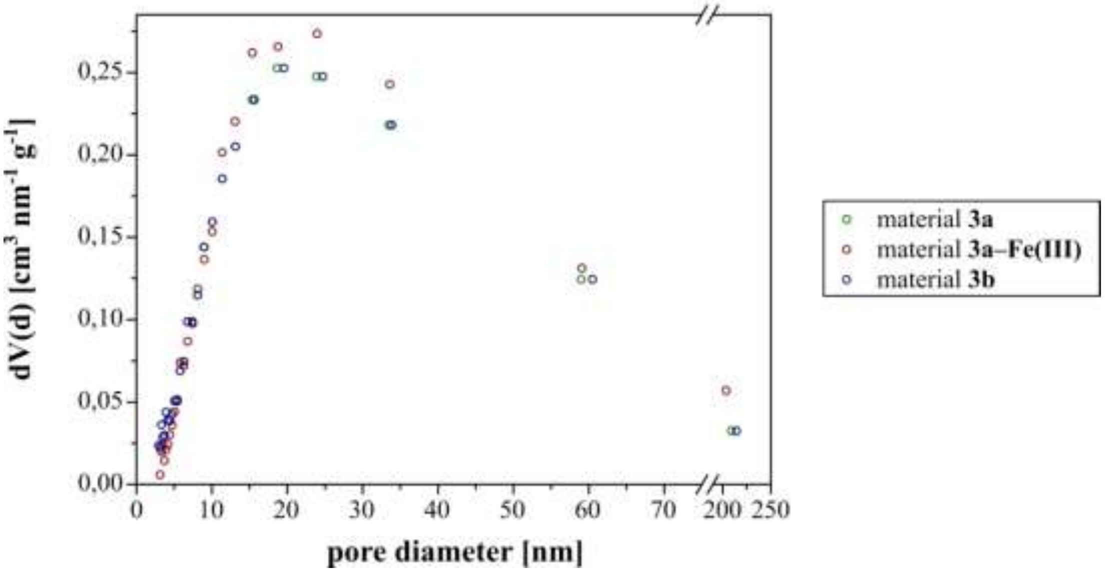

Figure S4. The pore size distribution of materials **3a** (green), **3b** (blue), and the complex material **3a**–Fe(III) (red)

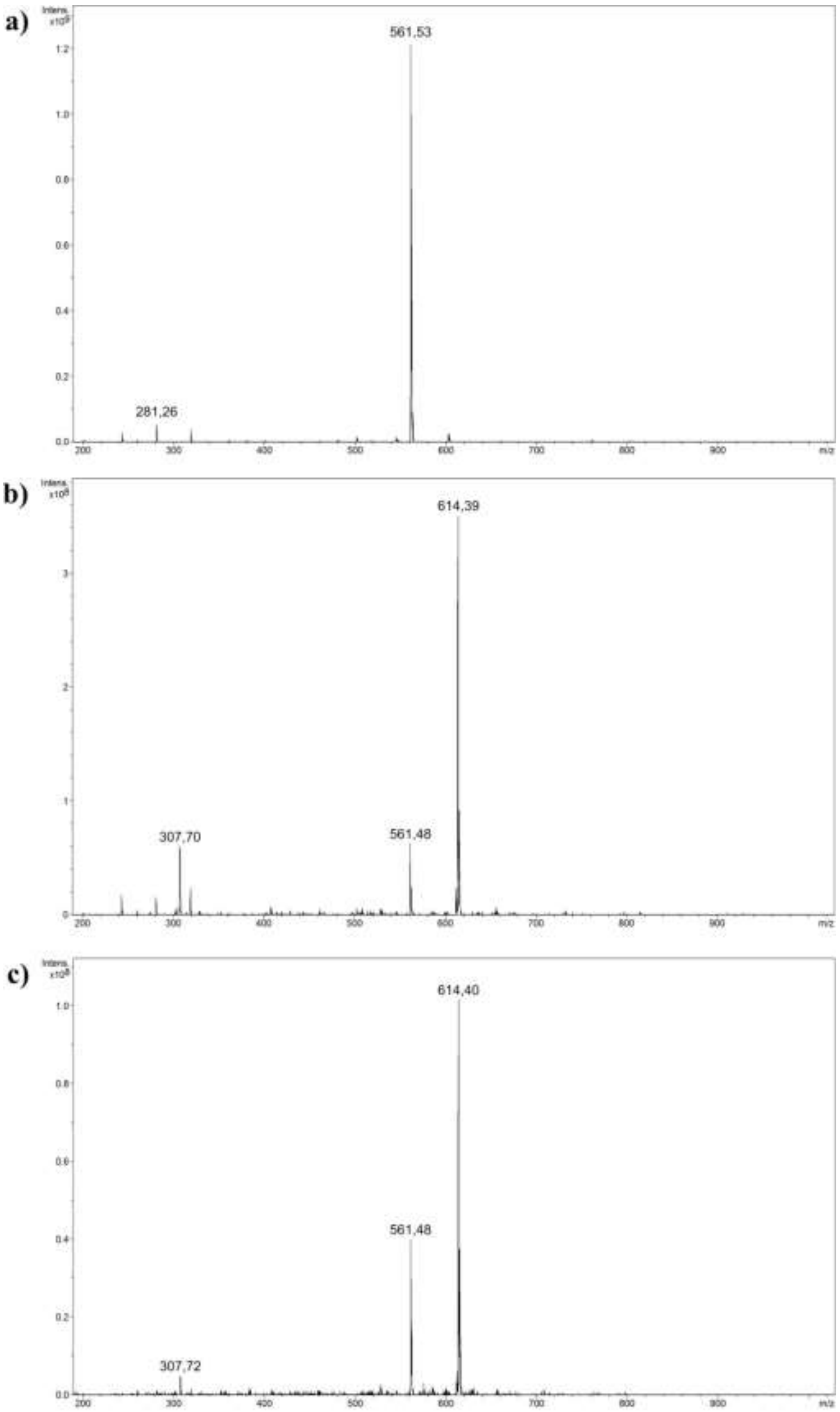

Figure S5. The ESI–MS spectra in positive mode of aqueous solutions: a) pure deferoxamine mesylate; b) deferoxamine–Fe(III) complex using $FeCl_3$; c) deferoxamine–Fe(II) complex using Mohr's salt ($(NH_4)_2Fe(SO_4)_2$)

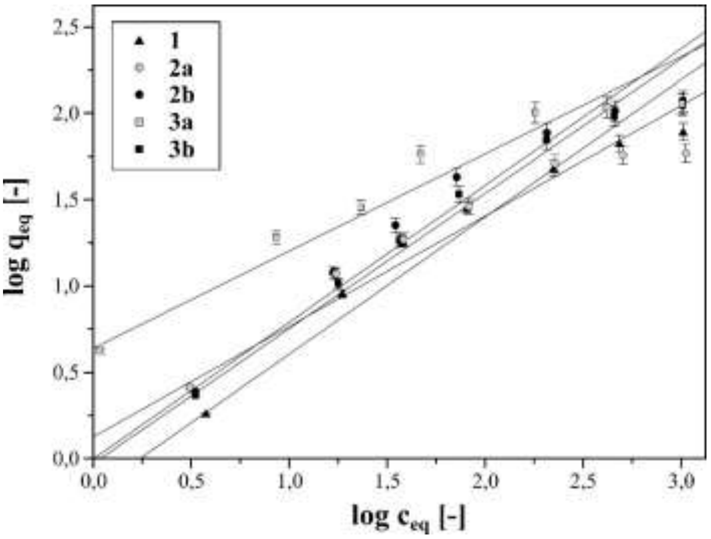

Figure S6. The experimental data fitting to the Freundlich isothermal model

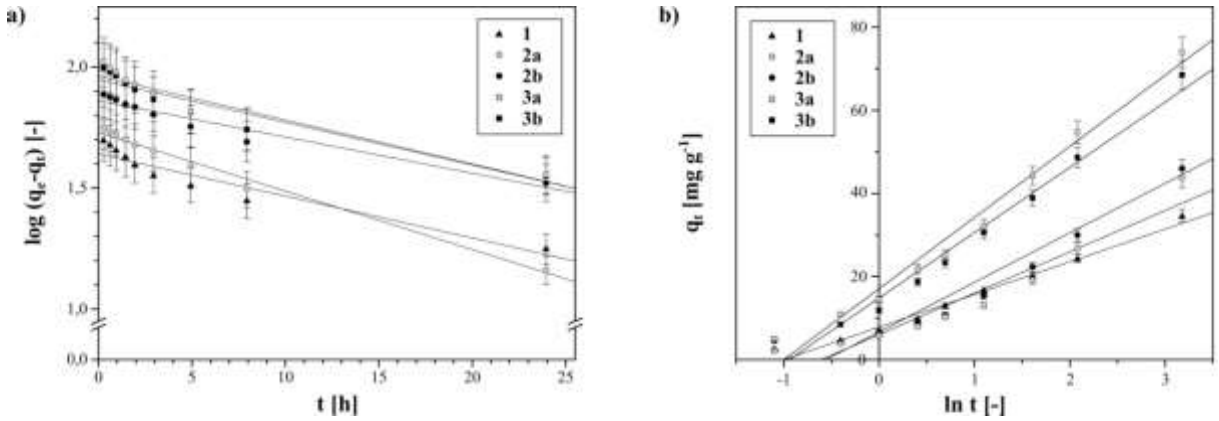

Figure S7. The kinetic experimental data fitted to: a) the pseudo–first–order kinetic model; b) the Elovich model

Table S1. Additional kinetic parameters calculated for Fe(III) adsorption kinetics fitted to the pseudo–first–order and the Elovich models

Material	Pseudo–first–order			Elovich model		
	$k_1 \cdot 10$ [h^{-1}]	k_i [mg g^{-1} h^{-1}]	R^2	α [mg g^{-1} min^{-1}]	β [mg g^{-1}]	R^2
**1**	0.40 ± 0.07	1.75 ± 0.45	0.9189	0.37 ± 0.02	0.129 ± 0.007	0.9809
**2a**	0.56 ± 0.07	2.99 ± 0.60	0.9875	0.32 ± 0.08	0.103 ± 0.011	0.9162
**2b**	0.34 ± 0.09	2.50 ± 0.84	0.9467	0.35 ± 0.07	0.095 ± 0.009	0.9339
**3a**	0.42 ± 0.09	3.86 ± 1.17	0.9044	0.78 ± 0.08	0.059 ± 0.004	0.9760
**3b**	0.41 ± 0.09	3.60 ± 1.11	0.9179	0.68 ± 0.08	0.064 ± 0.003	0.9747

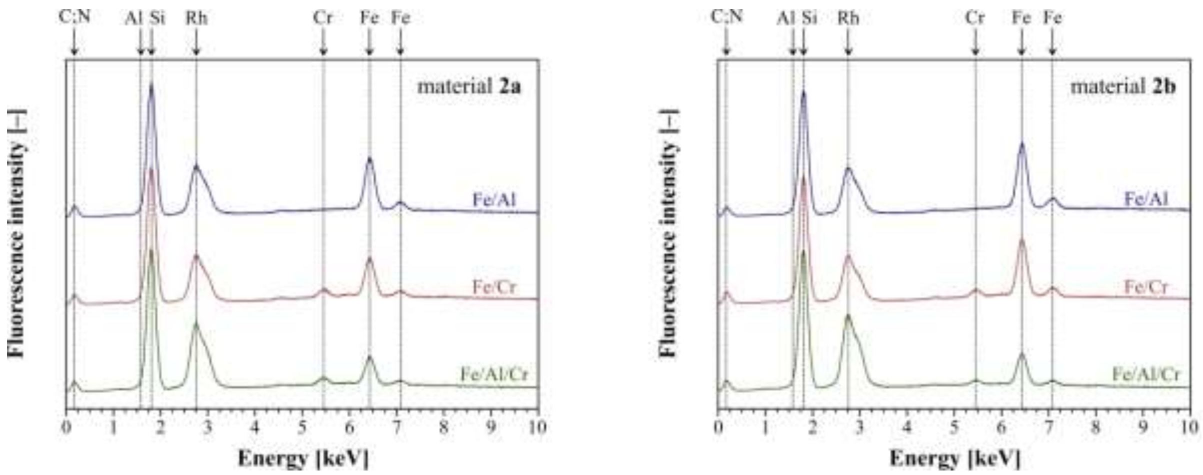

Figure S8. The XRF spectra of the complexes of materials **2a** and **2b** with trivalent metal cations. The materials were incubated with three different systems, containing Fe(III), Al(III), and/or Cr(III) cations

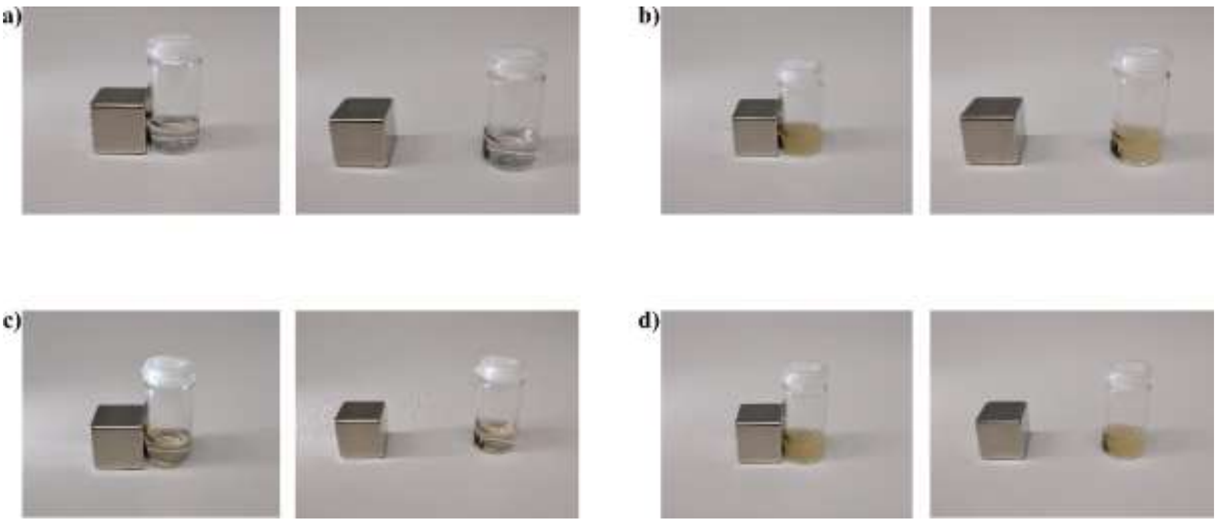

Figure S9. Concentrating of material **3a** (a, b) and **3b** (c, d) by neodymium magnet in: PBS buffer (a, c) and human serum (b, d). The distances between magnet and sample are 0 cm (left) and 5 cm (right)
